# Opioid growth factor receptor promotes adipose tissue thermogenesis via enhancing lipid oxidation

**DOI:** 10.1093/lifemeta/load018

**Published:** 2023-05-04

**Authors:** Shan Zhang, Jianhui Chen, Qingqing Li, Wenwen Zeng

**Affiliations:** Institute for Immunology, School of Medicine, Tsinghua-Peking Center for Life Sciences, Tsinghua University, Beijing 100084, China; Beijing Key Laboratory for Immunological Research on Chronic Diseases, Beijing 100084, China; Institute for Immunology, School of Medicine, Tsinghua-Peking Center for Life Sciences, Tsinghua University, Beijing 100084, China; Beijing Key Laboratory for Immunological Research on Chronic Diseases, Beijing 100084, China; Institute for Immunology, School of Medicine, Tsinghua-Peking Center for Life Sciences, Tsinghua University, Beijing 100084, China; Beijing Key Laboratory for Immunological Research on Chronic Diseases, Beijing 100084, China; Institute for Immunology, School of Medicine, Tsinghua-Peking Center for Life Sciences, Tsinghua University, Beijing 100084, China; Beijing Key Laboratory for Immunological Research on Chronic Diseases, Beijing 100084, China

**Keywords:** OGFr, thermogenesis, lipid metabolism, adipose tissue, diabetes

## Abstract

The thermogenic brown and beige adipocytes consume fatty acids and generate heat to maintain core body temperature in the face of cold challenges. Since their validated presence in humans, the activation of thermogenic fat has been an attractive target for treating obesity and related metabolic diseases. Here, we reported that the opioid growth factor receptor (*Ogfr*) was highly expressed in adipocytes and promoted thermogenesis. The mice with genetic deletion of *Ogfr* in adipocytes displayed an impaired capacity to counter environmental cold challenges. Meanwhile, *Ogfr* ablation in adipocytes led to reduced fatty acid oxidation, enhanced lipid accumulation, impaired glucose tolerance, and exacerbated tissue inflammation under chronic high-fat diet (HFD)-fed conditions. At the cellular level, OGFr enhanced the production of mitochondrial trifunctional protein subunit α (MTPα) and also interacted with MTPα, thus promoting fatty acid oxidation. Together, our study demonstrated the important role of OGFr in fatty acid metabolism and adipose thermogenesis.

## Introduction

The adipose tissues maintain metabolic homeostasis by coordinating energy storage and expenditure in response to caloric excess and deficit. Among the widely distributed fat pads, the thermogenic adipose tissues including brown and beige adipose tissues are mainly in charge of energy dissipation in the form of heat, whereas the white adipose tissues (WAT) are the major lipid storage sites [1]. Prompt mobilization and oxidation of the lipid supply are crucial for surviving environmental stress, such as environmental cold and food shortage. Adipose dysfunction contributes to diabetes and increases the risk of cardiovascular diseases among a plethora of associated health problems [2–5].

The thermogenic adipose tissues possess a high capacity to take up and utilize glucose and lipids [3]. In response to environmental cold, the thermogenic adipocytes are activated by sympathetic nerves through the signaling from the neurotransmitter norepinephrine (NE) to adrenergic receptors [1, 6, 7]. The glucose uptake and fatty acid oxidation are enhanced to fuel the mitochondria. Thermogenesis is then engaged via uncoupling substrate oxidation from ATP synthesis by uncoupling protein 1 (UCP1) on the inner mitochondrial membrane. The signaling pathway from NE to β3 adrenergic receptor (Adrb3) represents an intensively studied axis driving the thermogenic process in adipocytes. Interestingly, various processes mediated by additional receptors, which also facilitate energy expenditure, have been uncovered. For instance, the receptors for adenosine [8] and glucagon [9] activate adipose thermogenesis effectively; G protein-coupled receptor 3 (GPR3) is transcriptionally induced during cold exposure and drives thermogenesis via Gs-coupled activity in a ligand-independent manner [10]. The findings together implicate that adipocytes could adopt complex pathways in energy consumption, possibly dictated by the versatile collection of receptors.

Other than combating cold challenges, the thermogenic adipocytes also alter energy expenditure and shift energy balance in response to nutritional fluctuations. Feeding diets low in protein results in the activation of thermogenic fat [11]. High-fat diet (HFD) induces obesity and diabetes, and the thermogenic fats burn excess calories to counteract the metabolic consequences of obesity in mice though UCP1 is dispensable [12–16]. Cold-induced brown adipose tissue (BAT) activation has been found to inhibit tumor growth by regulating overall metabolic levels [17] and also overcoming visceral fat-associated obesity and diabetes [18]. Further, under the conditions of food shortage, the BAT activity is maintained, as evidenced by the increase in its cellularity and protein content during dietary restriction determined under thermoneutral conditions [19]. However, the signal events which may regulate the metabolic activities of brown and beige adipocytes and contribute to energy utilization in response to nutrient fluctuations are unclear [20].

The adipose tissues are composed of a diverse group of cell types including stromal cells, sympathetic nerves, and endothelial cells, in addition to the adipocytes [3, 21]. Intensive cell-to-cell communications have been recognized to mediate the tissue function. For instance, the vascular system communicates with other cell types to control adipocyte differentiation, function, and metabolism [22–27], and growth factors or microRNA from stromal vascular fraction (SVF) derived from adipose tissues regulates beige adipocyte differentiation and thermogenesis [24, 28–30]. Recent studies, particularly those utilizing the single-cell or single-nuclear RNA sequencing (scRNA-Seq or snRNA-Seq), have further implicated putative intercellular communications between the residential cell types which are potentially mediated by ligand-receptor pairs [31, 32]. When scrutinizing the receptors expressed in adipose tissues, we have noted that the opioid growth factor receptor (*Ogfr*) appears to be highly expressed and enriched in adipocytes despite being largely uncharacterized. Interestingly, the previously determined ligand for OGFr, methionine-enkephalin (MetEnk), belongs to the opioid family and is derived from its precursor proenkephalin (PENK) by cleavage [33–35]. Early researches have shown that MetEnk could regulate cell proliferation, but none of the known opioid receptors seem to mediate this effect [36, 37]. OGFr was originally identified as a new opioid receptor when mixing radio-labeled MetEnk with neuroblastoma homogenates [38]. Different from other opioid receptors which belong to the G protein-coupled receptor (GPCR) and are localized on the membrane, OGFr displays a random structure and is present both in the cytoplasm and nucleus [34]. The gene encoding OGFr has a broad expression profile in mouse and human tissues, and one of the characterized functions played by OGFr is to regulate cell proliferation [39–41]. Overall, it is unclear whether OGFr regulates adipocyte biology.

In adipose tissue, MetEnk has been reported to be produced from Group 2 innate lymphoid cells (ILC2s), an adipose residential immune cell type, and acts directly on adipocytes, promoting *Ucp1* expression and beige cell formation [42, 43]. Examination of the scRNA-Seq and snRNA-Seq datasets, however, reveals that the expression of *Penk* seems not to be restricted to ILC2s, but instead in various adipose stromal cells including adipocyte progenitor cells [44–46], suggesting that multiple cell types may participate in the ligand production and signaling. It is thus interesting to determine whether OGFr may play a significant role in adipocyte metabolism, and how MetEnk in stromal cells contributes to the energy balance.

In this study, we started by profiling the transcripts of receptors for neurotransmitters which may endow the responsiveness of adipocytes to external cues. We found that *Ogfr* was most highly expressed in adipocytes. We therefore generated mice with the conditional knockout allele of the *Ogfr* gene. Through adipocyte-specific deletion, we showed that *Ogfr*-deficient mice were defective in adipose thermogenesis during cold exposure. Immunoprecipitation further showed that OGFr interacted with mitochondrial trifunctional protein subunit α (MTPα), a key enzyme involved in fatty acid oxidation. The deletion of *Ogfr* resulted in lipid accumulation, impaired fatty acid oxidation, and increased susceptibility on glucose intolerance and tissue inflammation to HFD-induced obesity and diabetes. Furthermore, loss of *Penk* also led to hypothermia under cold challenge. Together, we have uncovered *Ogfr* as a crucial gene mediating lipid metabolism in adipocytes and offered a potential target for treating metabolic diseases.

## Results

### *Ogfr* is highly expressed in adipocytes and positively regulates energy consumption

**Figure 1:**
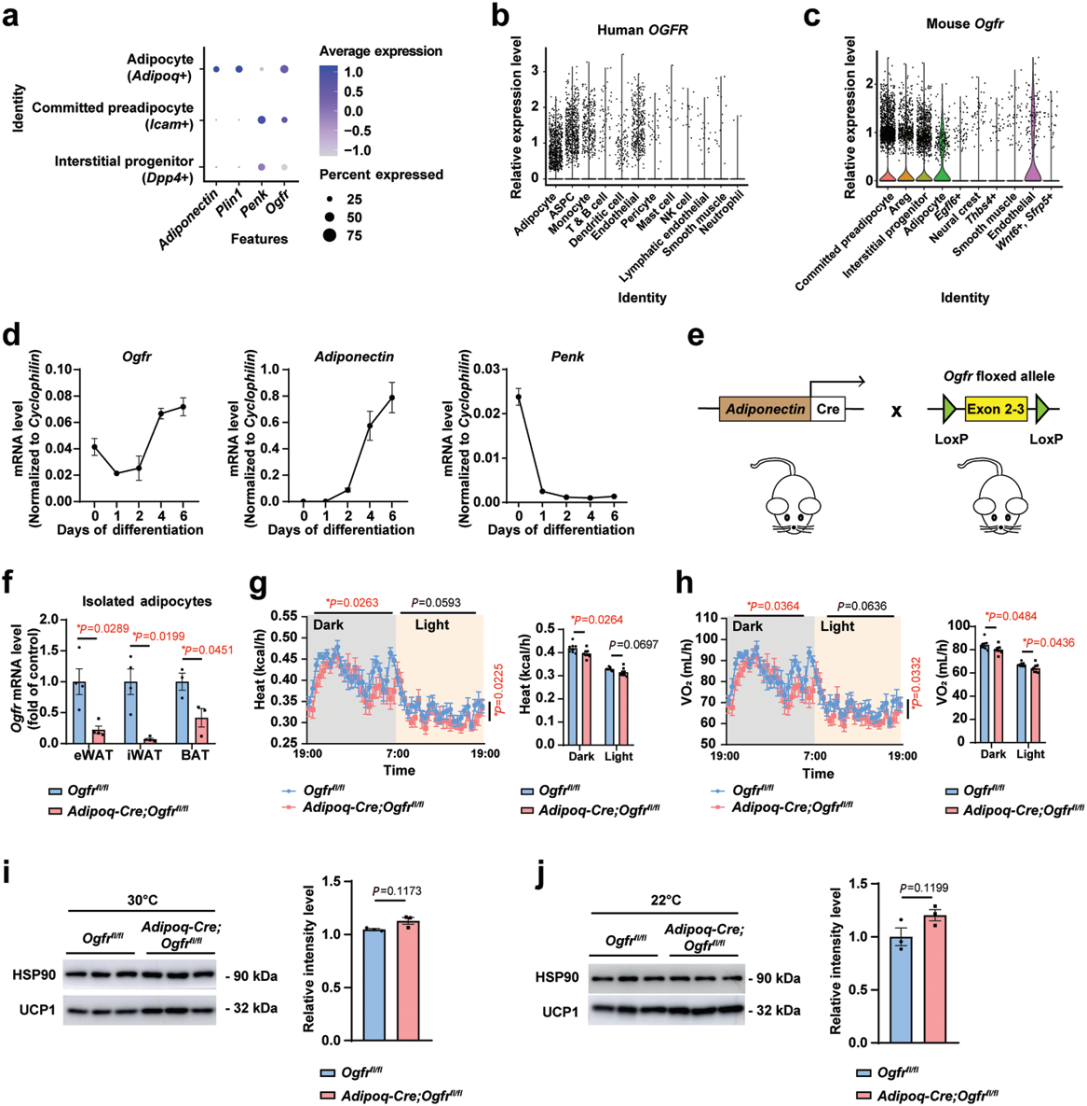
*Ogfr* is highly expressed in adipocytes and positively regulates energy consumption. (a) Analysis of single-cell gene expression profiles during mouse adipocyte differentiation. *Adipoq*, adiponectin; *Icam1*, intercellular adhesion molecule 1; *Dpp4*, dipeptidyl peptidase 4. (b) Individual gene t-SNE and violin plots showing the expression levels of *OGFR* from human snRNA-Seq results in different cell types in WAT. ASPC, adipose stem and progenitor cell. (c) Individual gene t-SNE and violin plots showing the expression levels of *Ogfr* from mouse scRNA-Seq results in different cell types in WAT. Areg, adipogenesis-regulatory cell; *Egfl6*, epidermal growth factor-like domain 6; *Thbs4*, thrombospondin 4; *Sfrp5*, secreted frizzled-related protein 5. (d) qPCR verification of *Ogfr*, *Adiponectin*, and *Penk* gene expression spanning differentiation of cultured control primary beige adipocytes. *n* = 3 per time point from female mice. Data are presented as mean ± SEM. (e) Strategy of *Ogfr*^*fl/fl*^ mice and generation of conditional knockout mice of *Adipoq-Cre;Ogfr*^*fl/fl*^. (f) qPCR verification of *Ogfr* expression in brown and white adipocytes isolated from *Adipoq-Cre;Ogfr*^*fl/fl*^ mice versus control littermates. eWAT, *n* = 4; iWAT, *n* = 4; BAT, *n* = 3. Female. Mean ± SEM; ^*^P < 0.05 by Student’s *t* test. (g) CLAMS metabolic cage analysis of heat production of *Adipoq-Cre;Ogfr*^*fl/fl*^ mice and control littermates housed at 22°C. *n* = 8. Female. Mean ± SEM; ^*^P < 0.05 by unmatched two-way ANOVA test for curve and Student’s *t* test for histogram. (h) CLAMS metabolic cage analysis of oxygen consumption of *Adipoq-Cre;Ogfr*^*fl/fl*^ mice and control littermates housed at 22°C. *n* = 8. Female. Mean ± SEM; ^*^P < 0.05 by unmatched two-way ANOVA test for curve, Student’s *t* test for histogram. (i) UCP1 immunoblot analysis of BAT from *Adipoq-Cre;Ogfr*^*fl/fl*^ mice and control littermates housing at 30°C. *n* = 3. Male. Mean ± SEM; Student’s *t* test. (j) UCP1 immunoblot analysis of BAT from *Adipoq-Cre;Ogfr*^*fl/fl*^ mice and control littermates housing at 22°C. *n* = 3. Female. Mean ± SEM; Student’s *t* test.

To explore new potential ligand-receptor intercellular signal axis in adipose tissues, we set out by analyzing the neurotransmitter receptors expressed in adipocytes. The scRNA-Seq results [47] show that *Ogfr* is among the most highly expressed receptors in adipocytes (Supplementary Fig. S1a), and its expression gradually increased during differentiation from progenitors to mature states in white adipocytes (Fig. 1a). In contrast, the ligand-encoding gene *Penk* gradually decreased during adipocyte differentiation (Fig. 1a). In both human snRNA-seq and mouse scRNA-seq of WAT [31, 47], *Penk* is largely restricted to the stromal cells which contain heterogeneous populations (Supplementary Fig. S1b and c), while *Ogfr* shows enrichment in mature adipocytes (Fig. 1b and c). We then examined the transcript levels of *Ogfr* and *Penk* by quantitative PCR (qPCR) through experimentally isolating mouse mature adipocytes and the SVF from interscapular BAT and inguinal and epididymal WAT (iWAT and eWAT). Consistently, *Ogfr* was highly expressed in adipocytes and *Penk* was enriched in the SVF (Supplementary Fig. S1d). When assessed in culture, *Ogfr* showed gradual upregulation during differentiation from progenitors to mature multilocular adipocytes resembling thermogenic beige adipocytes, and *Penk* was downregulated during differentiation (Fig. 1d), in agreement with the analysis from the scRNA-Seq dataset. Notably, the other members in opioid receptor family were expressed at much lower levels than *Ogfr* in both mouse and human adipocytes (Supplementary Fig. S1e), and *Ogfr* showed high expression levels in both WAT and BAT (Supplementary Fig. S1f).

To investigate whether OGFr may function to regulate adipocyte metabolic activities, we generated a mouse line with the conditional knockout alleles of *Ogfr* (*Ogfr*^*fl/fl*^) and crossed it to *Adiponectin-Cre* (*Adipoq-Cre*) mice to obtain adipocyte deletion (*Adipoq-Cre;Ogfr*^*fl/fl*^) (Fig. 1e). *Ogfr*^*fl/fl*^ mice served as the control animals in the following characterization. We examined the deletion efficiency in isolated adipocytes and the transcript level of *Ogfr* was reduced by 93% in beige adipocytes iWAT, 78% in white adipocytes of eWAT, and 58% in brown adipocytes in interscapular BAT from *Adipoq-Cre;Ogfr*^*fl/fl*^ mice (Fig. 1f).

When housed at room temperature (RT, 22°C), the *Adipoq-Cre;Ogfr*^*fl/fl*^ mice did not show significant differences in total body weight, food intake, or water intake except that the eWAT exhibited reduced fat mass compared with the control mice (Supplementary Fig. S2a and b). When examined by hematoxylin-eosin (HE) staining, the sizes of adipocytes in iWAT of the *Adipoq-Cre;Ogfr*^*fl/fl*^ mice were slightly larger than their counterparts in control mice (Supplementary Fig. S2c). We next measured whether the metabolic activities could be influenced using the metabolic cages. Interestingly, the *Adipoq-Cre;Ogfr*^*fl/fl*^ mice showed impaired ability for heat production (Fig. 1g) and reduced oxygen consumption (Fig. 1h) at the dark phase, whereas no significant changes in motility or respiratory exchange rate were observed compared with the control mice (Supplementary Figs S2d–f). The basal UCP1 protein levels in BAT from *Adipoq-Cre;Ogfr*^*fl/fl*^ mice and control littermates showed no obvious differences when housed at both thermoneutrality (TN, 30°C) and RT conditions (Fig. 1i and j). The results together showed that *Ogfr* is highly expressed in adipocytes and might positively regulate thermogenesis and energy consumption.

### OGFr promotes heat production by thermogenic fats in response to cold stress

To further investigate whether OGFr could affect the heat production ability for mice to counter environmental cold stress (4°C), we challenged the mice with acute cold exposure or chronic cold acclimation.

**Figure 2:**
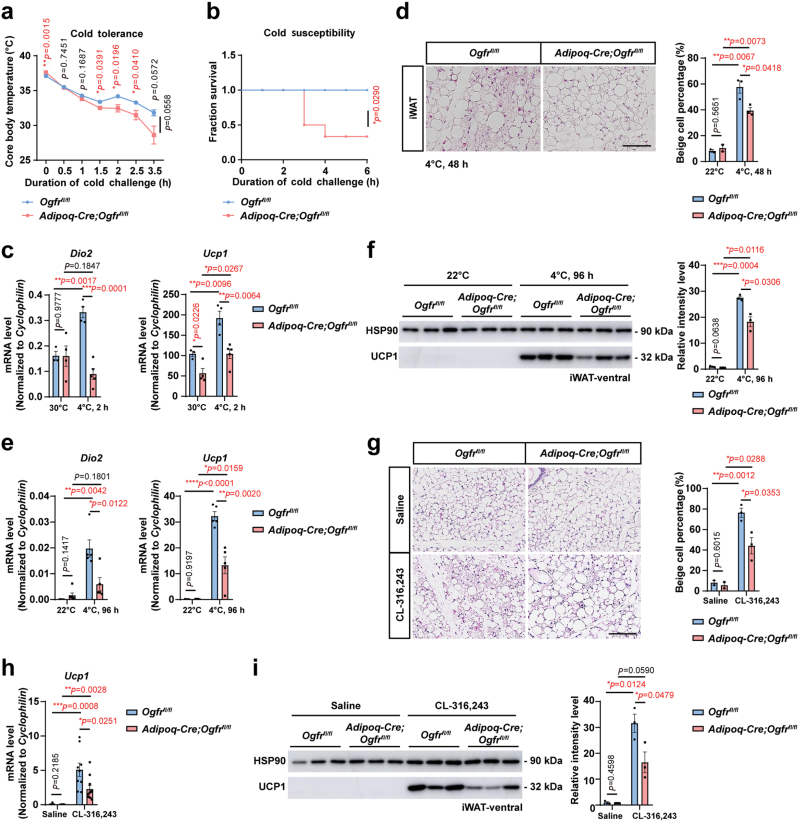
OGFr promotes heat production by thermogenic fats in response to cold stress. (a) Core body temperature of *Adipoq-Cre;Ogfr*^*fl/fl*^ mice versus control littermates under acute cold exposure from housing at 30°C for 10 days to 4°C. For *Adipoq-Cre;Ogfr*^*fl/fl*^, *n* = 4. For *Ogfr*^*fl/fl*^, *n* = 6. Male. Mean ± SEM; ^*^P < 0.05, ^**^P < 0.01 by Student’s *t* test, unmatched two-way ANOVA test for the curve. (b) Survival of *Adipoq-Cre;Ogfr*^*fl/fl*^ mice versus control littermates under acute cold exposure from housing at 30°C for 10 days to 4°C. For *Adipoq-Cre;Ogfr*^*fl/fl*^, *n* = 6. For *Ogfr*^*fl/fl*^, *n* = 5. Male. ^*^P < 0.05 by Mantel-Cox test. (c) qPCR analysis of *Dio2* and *Ucp1* gene expression changes in interscapular BAT from *Adipoq-Cre;Ogfr*^*fl/fl*^ mice versus control littermates cold challenged for 2 h after housing at 30°C for 10 days. For *Adipoq-Cre;Ogfr*^*fl/fl*^, *n* = 4 for 30°C and *n* = 5 for 4°C. For *Ogfr*^*fl/fl*^, *n* = 3 for 30°C and *n* = 4 for 4°C. Female. Mean ± SEM; ^*^P < 0.05, ^**^P < 0.01, ^***^P < 0.001, ^****^P < 0.0001 by Student’s *t* test. (d) HE staining and quantification of beige adipocyte percentage of iWAT from *Adipoq-Cre;Ogfr*^*fl/fl*^ mice and control littermates after cold challenge for 48 h at 4°C. Scale bar, 100 μm. For *Adipoq-Cre;Ogfr*^*fl/fl*^, *n* = 2 for 22°C and *n* = 3 for 4°C. For *Ogfr*^*fl/fl*^, *n* = 2 for 22°C and *n* = 3 for 4°C. Male. Mean ± SEM; ^*^P < 0.05, ^**^P < 0.01 by Student’s *t* test. (e) qPCR analysis of *Dio2* and *Ucp1* gene expression changes in iWAT from *Adipoq-Cre;Ogfr*^*fl/fl*^ mice versus control littermates housed at 22°C and cold challenged for 96 h at 4°C. For *Adipoq-Cre;Ogfr*^*fl/fl*^, *n* = 6 for 22°C and *n* = 5 for 4°C. For *Ogfr*^*fl/fl*^, *n* = 5 for 22°C and 4°C. Male. Mean ± SEM; ^*^P < 0.05, ^**^P < 0.01, ^***^P < 0.001, ^****^P < 0.0001 by Student’s *t* test. (f) UCP1 immunoblot analysis of iWAT from *Adipoq-Cre;Ogfr*^*fl/fl*^ mice and control littermates housed at 22°C and cold challenged for 96 h at 4°C. *n* = 3. Male. Mean ± SEM; ^*^P < 0.05, ^***^P < 0.001 by Student’s *t* test. (g) HE staining and quantification of beige adipocytes percentage of iWAT from *Adipoq-Cre;Ogfr*^*fl/fl*^ mice and control littermates with intraperitoneal saline or CL-316,243 treatment for 4 days at 22°C. Scale bar, 100 μm. For *Adipoq-Cre;Ogfr*^*fl/fl*^, *n* = 2 for saline group and *n* = 3 for CL-316,243 treated group. For *Ogfr*^*fl/fl*^, *n* = 2 for the saline group and *n* = 3 for CL-316,243 treated group. Female. Mean ± SEM; ^*^P < 0.05, ^**^P < 0.01 by Student’s *t* test. (h) qPCR analysis of *Ucp1* gene expression changes in iWAT from *Adipoq-Cre;Ogfr*^*fl/fl*^ mice versus control littermates with intraperitoneal saline or CL-316,243 treatment for 4 days at 22°C. For *Adipoq-Cre;Ogfr*^*fl/fl*^, *n* = 9 for the saline group and *n* = 10 for CL-316,243 treated group. For *Ogfr*^*fl/fl*^, *n* = 8 for saline group and *n* = 9 for CL-316,243 treated group. Female. Mean ± SEM; ^*^P < 0.05, ^**^P < 0.01, ^***^P < 0.001 by Student’s *t* test. (i) UCP1 immunoblot analysis of iWAT from *Adipoq-Cre;Ogfr*^*fl/fl*^ mice and control littermates with intraperitoneal saline or CL-316,243 treatment for 4 days at 22°C. *n* = 3. Female. Mean ± SEM; ^*^P < 0.05 by Student’s *t* test.

For acute cold stimulation, the animals housed at TN conditions were exposed to the low-temperature environment acutely, free access to water without food. Interestingly, the *Adipoq-Cre;Ogfr*^*fl/fl*^ mice displayed impaired ability to maintain core body temperature (Fig. 2a), which resulted in a higher mortality rate than the control mice (Fig. 2b). Accordingly, analysis of thermogenic gene expression showed decreased expression of iodothyronine deiodinase 2 (*Dio2*) and *Ucp1* in the BAT (Fig. 2c).

For chronic cold acclimation, the animals housed at RT conditions were changed to the cold environment for up to 96 h. When acclimated to the cold environment for 48 h, the *Adipoq-Cre;Ogfr*^*fl/fl*^ mice showed a reduction in the formation of multilocular beige adipocytes within iWAT examined by HE staining (Fig. 2d). The transcript levels of thermogenic genes including *Dio2* and *Ucp1* were lower in iWAT from *Adipoq-Cre;Ogfr*^*fl/fl*^ mice than those in the control mice after cold exposure for 96 h (Fig. 2e). Consistently, a reduced level of UCP1 protein was detected in iWAT of *Adipoq-Cre;Ogfr*^*fl/fl*^ mice compared with that of the control mice (Fig. 2f). We then stimulated the mice with Adrb3 agonist CL-316,243 to confirm the role of OGFr in thermogenesis. The *Adipoq-Cre;Ogfr*^*fl/fl*^ mice showed decreased ability to promote beige cell formation (Fig. 2g) and reduced expression of UCP1 at both RNA (Fig. 2h) and protein level (Fig. 2i) in iWAT after CL-316,243 stimulation for 4 days, supporting the conclusion that OGFr regulates thermogenesis in a NE-dependent manner. Together, the data suggest that the OGFr promotes heat generation by the thermogenic adipose tissues.

### OGFr enhances adipocyte lipid utilization for heat generation in response to cold exposure

**Figure 3:**
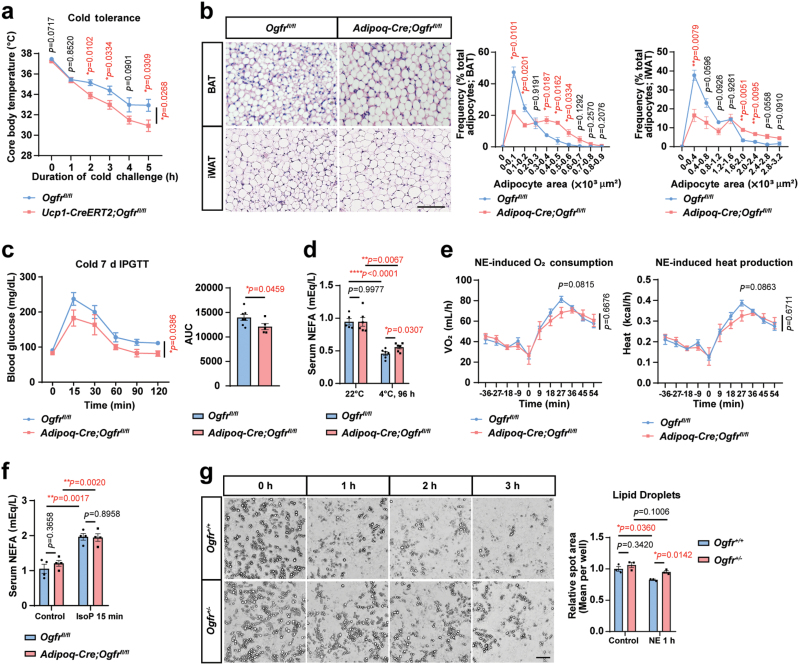
OGFr enhances adipocyte lipid utilization for heat generation in response to cold exposure. (a) Core body temperature of *Ucp1-CreERT2;Ogfr*^*fl/fl*^ mice versus control littermates under acute cold exposure from housing at 30°C to 4°C. *n* = 7. Female. Mean ± SEM; ^*^P < 0.05 by Student’s *t* test, unmatched two-way ANOVA test for curve. (b) HE staining and quantifications of interscapular BAT and iWAT from *Adipoq-Cre;Ogfr*^*fl/fl*^ mice and control littermates housed at 30°C. Scale bar, 100 μm. For iWAT, *n* = 3. For BAT, *n* = 4 for *Adipoq-Cre;Ogfr*^*fl/fl*^, *n* = 3 for *Ogfr*^*fl/fl*^. Female. Mean ± SEM; ^*^P < 0.05, ^**^P < 0.01 by Student’s *t* test. (c) IPGTT for *Adipoq-Cre;Ogfr*^*fl/fl*^ mice and control littermates after cold challenge for 7 days at 4°C. For *Adipoq-Cre;Ogfr*^*fl/fl*^, *n* = 4. For *Ogfr*^*fl/fl*^, *n* = 6. Female. Mean ± SEM; ^*^P < 0.05 by unmatched 2-way ANOVA test, Student’s *t* test for AUC. (d) Serum NEFA levels in *Adipoq-Cre;Ogfr*^*fl/fl*^ mice and control littermates housed at 22°C and cold challenged for 96 h at 4°C. For *Adipoq-Cre;Ogfr*^*fl/fl*^, *n* = 6 for 22°C and 4°C. For *Ogfr*^*fl/fl*^, *n* = 5 for 22°C and *n* = 6 for 4°C. Male. Mean ± SEM; ^*^P < 0.05, ^**^P < 0.01, ^****^P < 0.0001 by Student’s *t* test. (e) OCRs and heat production of anaesthetized *Adipoq-Cre;Ogfr*^*fl/fl*^ mice versus control littermates under NE stimulation at 22°C. *n* = 4. Male. Mean ± SEM; Student’s *t* test, unmatched two-way ANOVA test for curve. (f) *In vivo* lipolysis assay and serum NEFA levels in *Adipoq-Cre;Ogfr*^*fl/fl*^ mice and control littermates housed at 22°C and injected of isoproterenol for 15 min. *n* = 4. Female. Mean ± SEM; Student’s *t* test. (g) *In vitro* lipolysis analysis and quantified spot area of cultured primary adipocytes from *Ogfr*^*+/−*^ mice and control littermates, treated with 1 μmol/L NE for 0–3 h. *n* = 3. Female. Scale bar, 100 μm. Mean ± SEM; ^*^P < 0.05 by Student’s *t* test.

To validate the important role of OGFr in thermogenesis, we also crossed *Ogfr*^*fl/fl*^ to *Ucp1-CreERT2* mice generated in the early study [48] to delete *Ogfr* in thermogenic adipocytes by tamoxifen induction. Similarly, *Ucp1-CreERT2;Ogfr*^*fl/fl*^ presented no obvious changes in adipocyte sizes (Supplementary Fig. S3a), but lower core body temperature under acute cold challenges than the control mice (Fig. 3a).

To explore the underlying mechanisms of OGFr-regulated thermogenesis, we next characterized the energy consumption of *Adipoq-Cre;Ogfr*^*fl/fl*^ mice in response to environmental cold. When the mice were housed under TN conditions and fed with the normal chow diet, the body weight and fat mass did not show an obvious difference (Supplementary Fig. S3b). However, the adipocyte size was significantly larger in brown and beige adipocytes of *Adipoq-Cre;Ogfr*^*fl/fl*^ than in the control mice (Fig. 3b), indicating a lipid accumulation in *Ogfr*-deficient adipocytes.

Next, we performed the intraperitoneal glucose tolerance test (IPGTT) after the mice were exposed to the cold environment for 7 days, and the *Adipoq-Cre;Ogfr*^*fl/fl*^ mice showed an enhanced ability in glucose disposal from circulation (Fig. 3c). When the animals housed at RT conditions were exposed to the cold environment for 96 h, a higher level of serum non-esterified fatty acid (NEFA) was detected in *Adipoq-Cre;Ogfr*^*fl/fl*^ mice than the control mice (Fig. 3d), indicating a defect in oxidizing lipids for heat production.

Further, we detected NE-induced energy consumption *in vivo* using the metabolic cages. *Adipoq-Cre;Ogfr*^*fl/fl*^ mice showed the trend of reduced NE-induced oxygen consumption and heat production (Fig. 3e). We further examined whether the lipolysis differs after OGFr deletion and performed the lipolysis assay on both *in vivo* and in *in vitro* cultured adipocytes. We stimulated mice with isoproterenol to induce lipid dissipation and the released NEFA in the serum showed no obvious differences between *Adipoq-Cre;Ogfr*^*fl/fl*^ and control mice (Fig. 3f). The global knockout of *Ogfr* gene is lethal, therefore we crossed *CMV-Cre* to *Ogfr*^*fl/fl*^ and obtained the heterozygous *Ogfr* animals (*CMV-Cre;Ogfr*^*fl/+*^, *Ogfr*^*+/−*^) for *in vitro* characterization. When acutely stimulated with NE, adipocytes differentiated from *Ogfr*^*+/−*^ mice showed slower lipid dissipation rate in *Ogfr*-deficient cells compared to control adipocytes (Fig. 3g), and similar results were observed in adipocytes from *Adipoq-Cre;Ogfr*^*fl/fl*^ and control mice (Supplementary Fig. S3c). We also cultured primary adipocytes from *Adipoq-Cre;Ogfr*^*fl/fl*^ and control mice and collected the protein samples during differentiation at 0, 1, 2, 4, and 6 days. The immunoblot indicated that the cells from *Adipoq-Cre;Ogfr*^*fl/fl*^ mice showed reduced perilipin1 (PLIN1) levels compared to control cells (Supplementary Fig. S3d). We then stained the mature adipocytes with oil red followed by extraction with isopropyl alcohol and measurement with absorbance at OD510 nm. A reduced level of lipid was detected in cells from *Adipoq-Cre;Ogfr*^*fl/fl*^ mice (Supplementary Fig. S3e). Collectively, our data showed that loss of OGFr might impair adipocyte differentiation, which is consistent with the role of MetEnk in regulating cell proliferation.

We further examined whether NE-mediated lipolysis pathway differs in *Ogfr*^*+/−*^ and control adipocytes. Under NE stimulation, the phosphorylation of hormone-sensitive lipase (HSL) was induced but no obvious differences were observed between *Ogfr*^*+/−*^ and control cells (Supplementary Fig. S3f), indicating that the slower lipid dissipation might be attributed to reduced oxidation downstream of lipolysis. Collectively, the results showed that OGFr promotes lipid utilization for thermogenic adipose tissues downstream of NE-mediated lipolytic pathway.

### OGFr interacts with MTPα

To further investigate how OGFr regulates thermogenesis and lipid utilization, we generated the C-terminal HA-tagged OGFr mouse (OGFr-HA) and identified the molecular partners interacting with OGFr. We performed affinity purification of HA-tagged OGFr using anti-HA magnetic beads in iWAT and the immunopurified proteins were analyzed by mass spectrum (Supplementary Fig. S4a). A list of proteins was revealed by this approach, including MTPα and MTPβ which are involved in the fatty acid oxidation pathway (Supplementary Fig. S4b).

**Figure 4:**
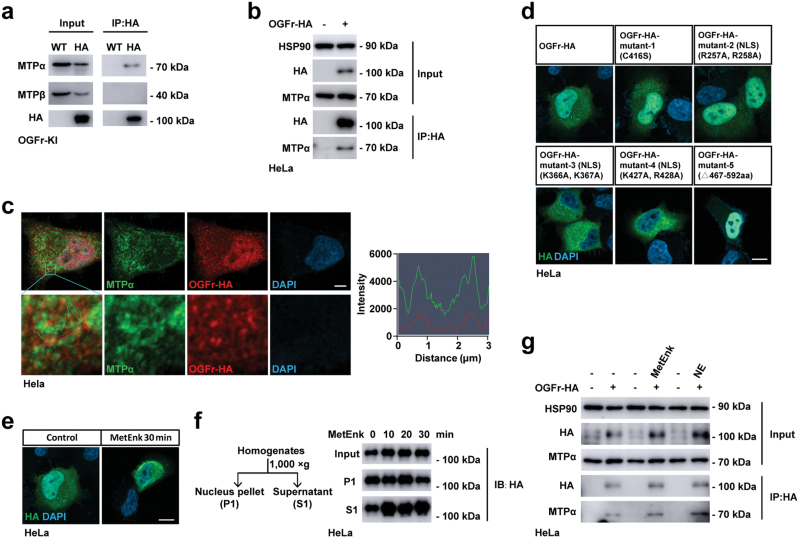
OGFr interacts with MTPα. (a) Immunoblot analysis of MTPα, MTPβ, and HA following immunoprecipitation with anti-HA in iWAT from OGFr-HA KI and control mice. (b) Immunoblot analysis of MTPα, HA, and HSP90 following immunoprecipitation with anti-HA in OGFr-HA overexpressed HeLa cells. (c) Confocal images of immunohistochemistry of OGFr-HA and MTPα in OGFr-HA-overexpressing HeLa cells. Scale bar, 5 μm. (d) Confocal images of immunohistochemistry of OGFr-HA and mutants in OGFr-HA or mutants-overexpressing HeLa cells. Scale bar, 10 μm. (e) Confocal images of immunohistochemistry of OGFr-HA in OGFr-HA-overexpressing HeLa cells under 1 μg/mL MetEnk stimulation for 30 min. Scale bar, 10 μm. (f) Immunoblot analysis of HA by cell fractionation of OGFr-HA-overexpressing HeLa cells under 1 μg/mL MetEnk stimulation for 0–30 min. P1 for nucleus pellet and S1 for supernatant, centrifuged at 1000 × *g* for 5 min. (g) Immunoblot analysis of MTPα, HA, and HSP90 in input or eluates following immunoprecipitation with anti-HA in OGFr-HA-overexpressing HeLa cells under 1 μg/mL MetEnk or 0.1 μmol/L NE stimulation for 30 min.

To verify the interaction, we then performed anti-HA immunoprecipitation of OGFr-HA in iWAT from OGFr-HA mice (Fig. 4a) and also OGFr-HA expressed in HeLa cells (Fig. 4b). Immunoblot analysis of input and eluates showed enrichment of endogenous MTPα following anti-HA immunoprecipitation. Next, we carried out the immunofluorescence-based analysis of OGFr and MTPα localization in HeLa cells and the imaging showed that a fraction of OGFr colocalized with MTPα (Fig. 4c), confirming the interaction between OGFr and MTPα. Also, when overexpressing OGFr and MTPα in cultured HEK293T cells, we detected increased levels of MTPα protein and mRNA (Supplementary Fig. S4c and d). *Vice versa*, reduced protein level of MTPα was observed in *Adipoq-Cre;Ogfr*^*fl/fl*^ mice versus control mice after cold challenge or CL-316,243 stimulation (Supplementary Fig. S4e and f), indicating that OGFr promotes the production of MTPα. MTPα is the rate-limiting enzyme involved in the fatty acid oxidation and catalyzes the last three steps of mitochondrial beta-oxidation of long-chain fatty acids [49, 50]. The enhanced production of MTPα by OGFr and the interaction between OGFr and MTPα indicated that OGFr might positively regulate the fatty acid oxidation process.

Unlike the classical opioid receptors which are all GPCRs, OGFr contains nuclear localization sequences and could exist both in the nucleus and cytoplasm [34]. When nuclear localization signals were mutated, OGFr tended to accumulate in the cytoplasm and the C-terminal repeated domains seemed to be also crucial for the nuclear localization (Fig. 4d), which was consistent with the previous study performed in COS-7 monkey kidney cells [51]. To determine the possibility that MetEnk may affect OGFr function by regulating its localization, we treated the OGFr-HA-expressing HeLa cells with MetEnk and analyzed the localization of OGFr by immunofluorescence and subcellular fractionation. The results showed that the distribution of OGFr in the cytoplasm increased after MetEnk stimulation (Fig. 4e and f). We further performed anti-HA immunoprecipitation using OGFr-HA-expressing HeLa cells in the absence or presence of MetEnk or NE to explore whether MetEnk and NE regulate the interaction between OGFr and MTPα. Interestingly, MetEnk and NE treatment resulted in enhanced interaction between OGFr and MTPα (Fig. 4g), indicating that NE-stimulated lipolysis may supply substrates to fuel the fatty acid oxidation, which subsequently facilitated the complex formation. Together, we showed that MetEnk could enhance the subcellular localization of OGFr in the cytoplasm, thereby promoting its interaction with MTPα and lipid oxidation.

### OGFr enhances adipocyte fatty acid oxidation

During prolonged starvation, the animals are obliged to shift from carbohydrate metabolism to fat metabolism [52, 53]. The fatty acid oxidation could be stimulated when glucose levels become low and fasting doubles the rate of fatty acid oxidation which is required for the maintenance of body temperature [54–57].

**Figure 5:**
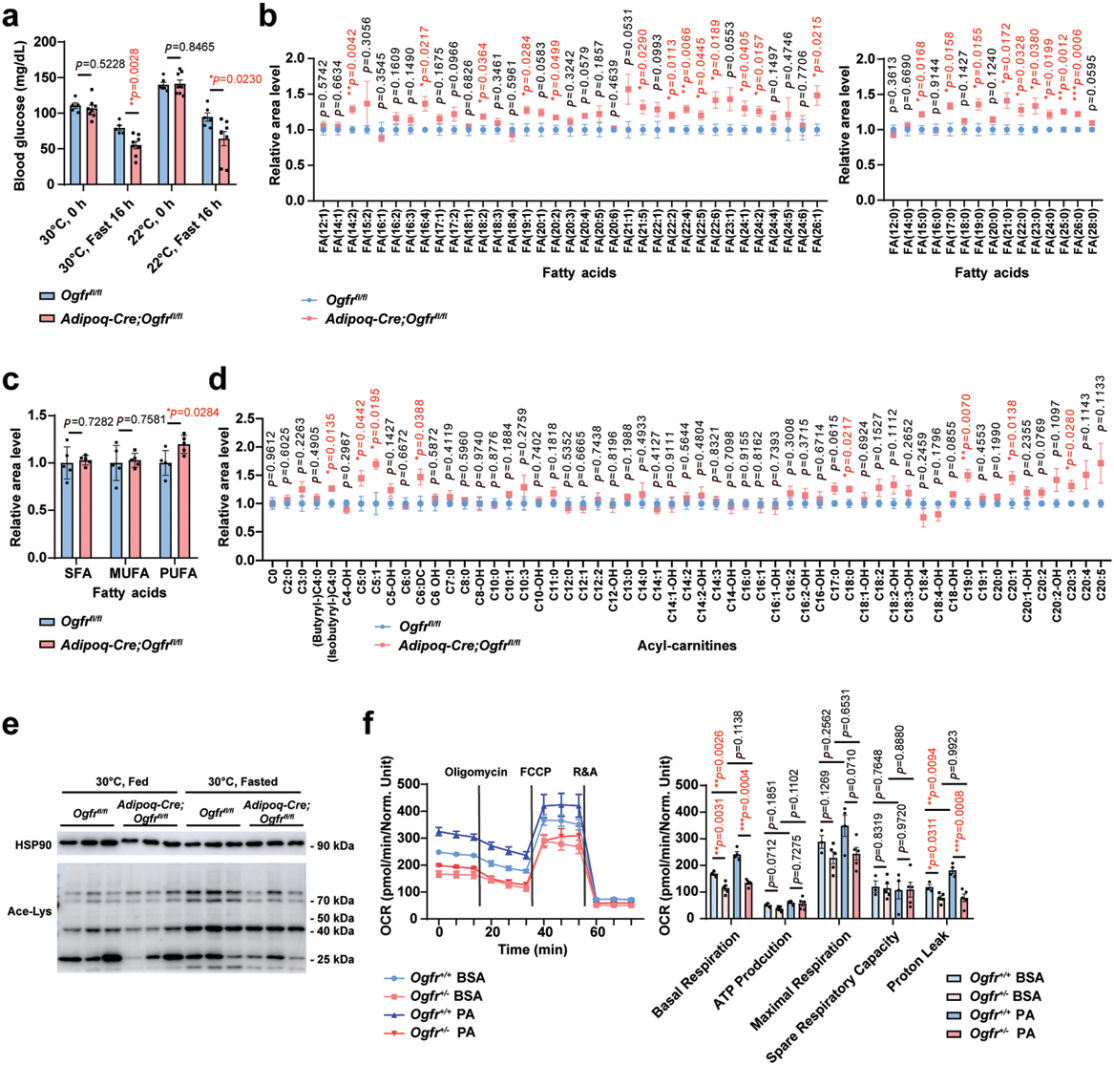
OGFr enhances adipocyte fatty acid oxidation. (a) Blood glucose levels in *Adipoq-Cre;Ogfr*^*fl/fl*^ mice and control littermates before and after fasting for 16 h housed at 30°C and 22°C. For *Adipoq-Cre;Ogfr*^*fl/fl*^, *n* = 8 for 30 and 22°C. For *Ogfr*^*fl/fl*^, *n* = 5 for 30°C and *n* = 6 for 22°C. Female. Mean ± SEM; ^*^P < 0.05, ^**^P < 0.01 by Student’s *t* test. (b) Serum fatty acid profile of *Adipoq-Cre;Ogfr*^*fl/fl*^ mice and control littermates after fasting for 24 h housed at 30°C. *n* = 5. Male. Mean ± SEM; ^*^P < 0.05, ^**^P < 0.01, ^***^P < 0.001 by Student’s *t* test. (c) Serum fatty acid levels of *Adipoq-Cre;Ogfr*^*fl/fl*^ mice and control littermates after fasting for 24 h housed at 30°C. SFA, saturated fatty acid. MUFA, monounsaturated fatty acid. PUFA, polyunsaturated fatty acid. *n* = 5. Male. Mean ± SEM; ^*^P < 0.05, ^**^P < 0.01, ^***^P < 0.001 by Student’s *t* test. (d) Serum acyl-carnitine profile of *Adipoq-Cre;Ogfr*^*fl/fl*^ mice and control littermates after fasting for 24 h housed at 30°C. *n* = 5. Male. Mean ± SEM; ^*^P < 0.05, ^**^P < 0.01 by Student’s *t* test. (e) Ace-Lys immunoblot analysis of interscapular BAT from *Adipoq-Cre;Ogfr*^*fl/fl*^ mice and control littermates housed at 30°C, fasted for 16 h or fed. *n* = 3. Female. (f) OCR analysis of cultured primary adipocytes from *Ogfr*^*+/−*^ mice and control littermates. Treated with BSA or 200 μmol/L PA, followed with oligomycin (1 μmol/L), FCCP (1 μmol/L), rotenone (1 μmol/L), and antimycin A (2 μmol/L) as indicated. For *Ogfr*^*+/−*^ cells, *n* = 5 for BSA group and PA group. For control cells, *n* = 3 for BSA group and *n* = 4 for PA group. Female. Mean ± SEM; ^*^P < 0.05, ^**^P < 0.01, ^***^P < 0.001 by Student’s *t* test.

We next determined whether OGFr may affect the fatty acid oxidation during nutrient deprivation of mice. Upon food restriction for 16 h, we detected a faster decline of blood glucose in *Adipoq-Cre;Ogfr*^*fl/fl*^ mice than in the control mice both under TN and RT conditions (Fig. 5a). The liquid chromatography coupled to mass spectrum (LC-MS/MS) was used to quantify fatty acids in serum samples from *Adipoq-Cre;Ogfr*^*fl/fl*^ and control animals after fasting for 24 h. Indeed, most NEFA lipid species in *Adipoq-Cre;Ogfr*^*fl/fl*^ serum were significantly higher post fasting (Fig. 5b and c), in support of the crucial role of OGFr in promoting lipid consumption.

Fatty acid species are converted to acyl-CoA esters followed by transportation in the form of acyl-carnitines into the mitochondrial matrix to undergo the fatty acid oxidation process [49]. Unutilized substrates subsequently enter the blood circulation or urine and the diagnosis of fatty acid oxidation disorders can be commonly achieved by detecting the acyl-carnitine profile in the blood [58]. We then adopted LC-MS/MS to quantify acyl-carnitines in serum samples from *Adipoq-Cre;Ogfr*^*fl/fl*^ and control mice. Serum acyl-carnitines especially long-chain acyl-carnitines were significantly higher in *Adipoq-Cre;Ogfr*^*fl/fl*^ mice post fasting (Fig. 5d), further confirming the defects of fatty acid oxidation in *Ogfr*-deficient mice.

Energy deficit leads to elevation of lysine acetylation, a form of post-translational protein modification, on various enzymes involved in metabolic processes such as fatty acid oxidation, which is positively correlated with their activity [59–61]. We next examined the Acetylated-Lysine (Ace-Lys) levels in BAT before and after fasting at TN conditions, and found that the *Adipoq-Cre;Ogfr*^*fl/fl*^ mice showed reduced levels of Ace-Lys compared to control mice (Fig. 5e).

We also measured the oxygen consumption rate (OCR) and extracellular acidification rate (ECAR) in cultured adipocytes from iWAT with reduced expression of *Ogfr* to examine the fatty acid oxidation process *in vitro*. When given palmitic acids (PA), the adipocytes from *Ogfr*^*+/−*^ mice showed defective cellular respiration rate and proton leak (Fig. 5f) but increased glycolytic reserve when given glucose (Supplementary Fig. S5), consistent with impaired fatty acid oxidation upon OGFr reduction.

Together, our data showed that OGFr enhances fatty acid oxidation and utilization, resulting in the reduction of long-chain fatty acids and acyl-carnitines in circulation.

### OGFr protects from HFD-induced glucose intolerance

**Figure 6:**
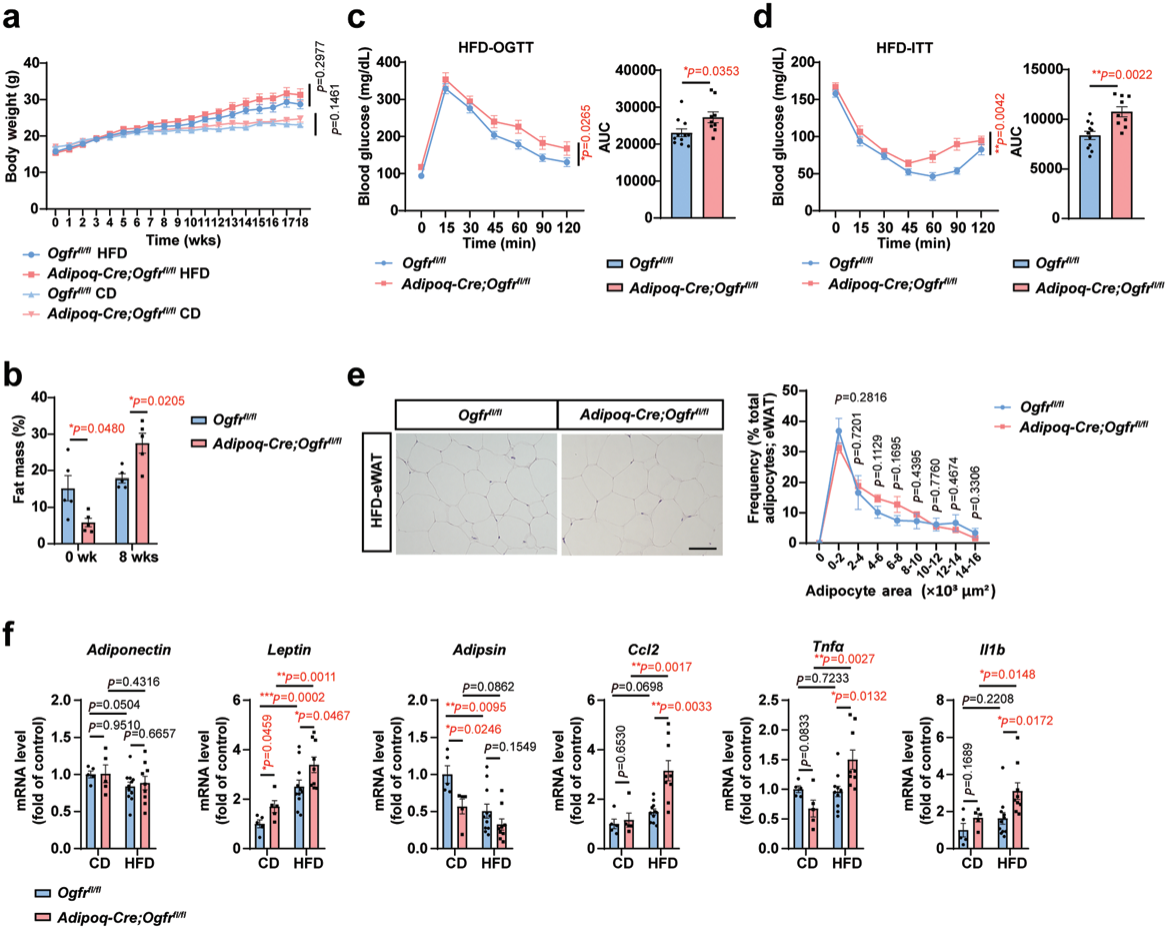
OGFr protects from HFD-induced glucose intolerance. (a) Body weight gain of *Adipoq-Cre;Ogfr*^*fl/fl*^ mice and control littermates fed chow diet or HFD at 22°C. For *Adipoq-Cre;Ogfr*^*fl/fl*^, *n* = 5 for chow diet and *n* = 9 for HFD. For *Ogfr*^*fl/fl*^, *n* = 5 for chow diet and *n* = 11 for HFD. CD, chow diet. HFD, high-fed diet. Female. Mean ± SEM; unmatched two-way ANOVA test for curve. (b) Fat mass (normalized to body weight) of *Adipoq-Cre;Ogfr*^*fl/fl*^ mice and control littermates fed HFD for 8 weeks at 22°C. *n* = 5. Male. Mean ± SEM; ^*^P < 0.05 by Student’s *t* test. (c) OGTT for *Adipoq-Cre;Ogfr*^*fl/fl*^ mice and control littermates fed HFD for 15 weeks at 22°C. For *Adipoq-Cre;Ogfr*^*fl/fl*^, *n* = 9. For *Ogfr*^*fl/fl*^, *n* = 11. Female. Mean ± SEM; ^*^P < 0.05 by two-way ANOVA test, Student’s *t* test for AUC. (d) ITT for *Adipoq-Cre;Ogfr*^*fl/fl*^ mice and control littermates fed HFD for 16 weeks at 22°C. For *Adipoq-Cre;Ogfr*^*fl/fl*^, *n* = 9. For *Ogfr*^*fl/fl*^, *n* = 11. Female. Mean ± SEM; ^**^P < 0.01 by two-way ANOVA test, Student’s *t* test for AUC. (e) HE staining and quantifications of eWAT from *Adipoq-Cre;Ogfr*^*fl/fl*^ mice and control littermates fed HFD for 18 weeks at 22°C. Scale bar, 100 μm. For *Adipoq-Cre;Ogfr*^*fl/fl*^, *n* = 3. For *Ogfr*^*fl/fl*^, *n* = 4. Female. Mean ± SEM; Student’s *t* test. (f) qPCR determination of adipokines, inflammatory gene transcripts in eWAT from *Adipoq-Cre;Ogfr*^*fl/fl*^ mice versus control littermates fed chow diet or HFD for 18 weeks at 22°C. For *Adipoq-Cre;Ogfr*^*fl/fl*^, *n* = 5 for chow diet and *n* = 9 for HFD. For *Ogfr*^*fl/fl*^, *n* = 5 for chow diet and *n* = 11 for HFD. CD, chow diet. HFD, high-fed diet. Female. Mean ± SEM; ^*^P < 0.05, ^**^P < 0.01, ^***^P < 0.001 by Student’s *t* test.

Chronic nutrition surplus causes excessive accumulation of lipids. The defective fatty acid oxidation and thermogenesis may aggravate the development of obesity, diabetes, and tissue inflammation [62, 63]. Therefore, we determined whether OGFr affects energy balance against nutrient overload. Though the total body weight did not differ significantly between *Adipoq-Cre;Ogfr*^*fl/fl*^ and control mice (Fig. 6a), the *Adipoq-Cre;Ogfr*^*fl/fl*^ mice showed increased fat mass after HFD feeding for 8 weeks (Fig. 6b). To determine whether OGFr-regulated adiposity affectes glucose homeostasis and insulin sensitivity, we performed the oral glucose tolerance test (OGTT) and insulin tolerance test (ITT). The *Adipoq-Cre;Ogfr*^*fl/fl*^ displayed impaired systemic glucose tolerance (Fig. 6c) and worsened insulin sensitivity (Fig. 6d). Meanwhile, the eWAT showed increased lipid accumulation in *Adipoq-Cre;Ogfr*^*fl/fl*^ mice (Fig. 6e). Elevated fat inflammation was also observed, as the proinflammatory genes such as *Tnfα*, *Ccl2*, and *Il1b* were upregulated in eWAT of *Adipoq-Cre;Ogfr*^*fl/fl*^ mice (Fig. 6f and Supplementary Fig. S6). The expression of *leptin* was higher in *Adipoq-Cre;Ogfr*^*fl/fl*^ mice (Fig. 6f), further supporting the increased adiposity in the *Ogfr*-deficient condition [64]. Overall, our results showed that OGFr positively regulates lipid utilization during energy surplus and thereby influences adiposity, glucose tolerance, insulin sensitivity, and tissue inflammation.

### Stromal MetEnk to adipocyte OGFr axis enhances thermogenesis in response to cold exposure

**Figure 7:**
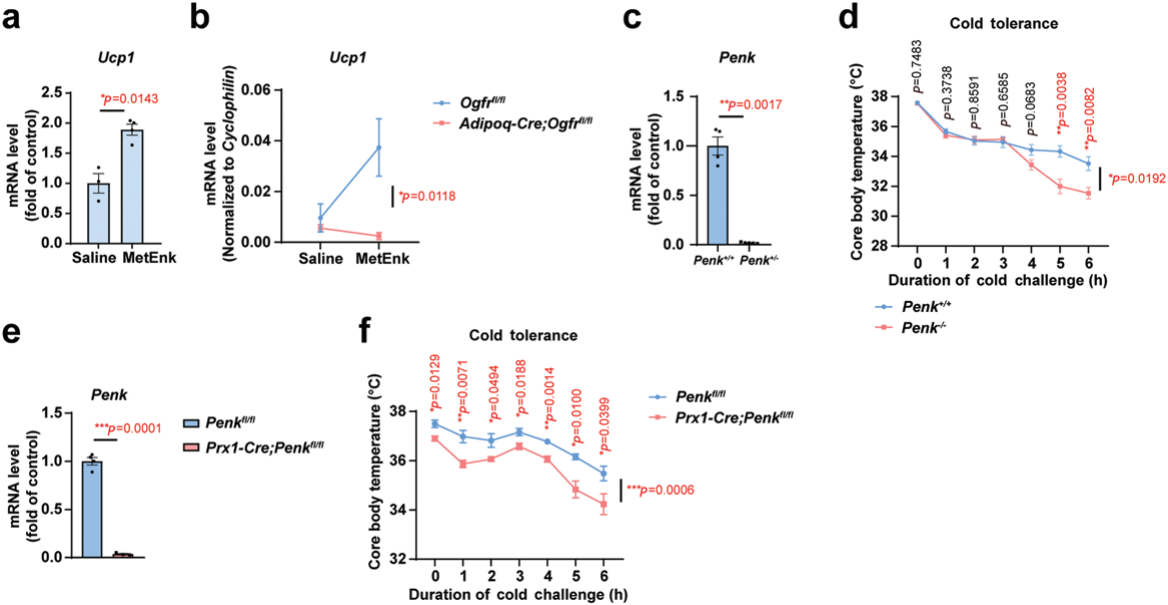
Stromal MetEnk to adipocyte OGFr axis enhances thermogenesis in response to cold exposure. (a) qPCR verification of *Ucp1* expression changes in interscapular BAT from control mice treated with 10 mg/kg MetEnk to the dorsal nuchal region for 4 days housed at 30°C. For MetEnk group, *n* = 4. For saline group, *n* = 3. Male. Mean ± SEM; ^*^P < 0.05 by Student’s *t* test. (b) qPCR analysis of *Ucp1* gene expression changes in iWAT from *Adipoq-Cre;Ogfr*^*fl/fl*^ mice versus control littermates with intraperitoneal saline or 10 mg/kg MetEnk treatment for 4 days at 22°C. For *Adipoq-Cre;Ogfr*^*fl/fl*^, *n* = 6. For *Ogfr*^*fl/fl*^, *n* = 4. Female. Mean ± SEM; ^*^P < 0.05 by unmatched two-way ANOVA test. (c) qPCR verification of *Penk* expression in interscapular BAT from *Penk*^*−/−*^ mice and control littermates housed at 30°C. For *Penk*^*−/−*^, *n* = 5. For control mice, *n* = 4. Male. Mean ± SEM; ^**^P < 0.01 by Student’s *t* test. (d) Core body temperature test of *Penk*^*−/−*^ mice versus control littermates under acute cold exposure from housing at 30°C for 10 days to 4°C. *n* = 6. Female. Mean ± SEM; ^*^P < 0.05, ^**^P < 0.01 by Student’s *t* test, unmatched two-way ANOVA test for the curve. (e) qPCR verification of *Penk* expression in iWAT from *Prx1-Cre;Penk*^*fl/fl*^ mice and control littermates housed at 30°C. For *Prx1-Cre;Penk*^*fl/fl*^ mice, *n* = 3. For control mice, *n* = 4. Female. Mean ± SEM; ^***^P < 0.001 by Student’s *t* test. (f) Core body temperature test of *Prx1-Cre;Penk*^*fl/fl*^ mice versus control littermates under acute cold exposure from housing at 30°C for 10 days to 4°C. For *Prx1-Cre;Penk*^*fl/fl*^ mice, *n* = 6. For control mice, *n* = 5. Female. Mean ± SEM; ^*^P < 0.05, ^**^P < 0.01, ^***^P < 0.001 by Student’s *t* test, unmatched two-way ANOVA test for curve.

Lastly, we validated the role of OGFr ligand, MetEnk, in mediating the thermogenic process in BAT. Previous study has shown that MetEnk promotes beige cell formation in iWAT [42]. We found that *in vivo* delivery of MetEnk peptides into the BAT region under TN conditions led to the increase of the mRNA level of *Ucp1* in BAT (Fig. 7a). Treatment with MetEnk peptides also increased *Ucp1* expression in iWAT under RT conditions and the effect was abrogated in *Adipoq-Cre;Ogfr*^*fl/fl*^ mice (Fig. 7b). Further, we generated the *Penk* knockout (*Penk*^*−/−*^) mice (Fig. 7c) and found that *Penk* ablation impaired the capacity of the mice to maintain core body temperature under acute cold challenge (Fig. 7d), despite undiscernible changes in lipid accumulation in BAT and iWAT under TN conditions (Supplementary Fig. S7a). We next determined the role of stromal cell-derived MetEnk in regulating adipocyte thermogenesis. The expression of *Penk* can be largely abrogated in iWAT when crossing *Penk*^*fl/fl*^ with *Prx1-Cre* mice which drove deletion of *Penk* in adipose stromal cells (*Prx1-Cre;Penk*^*fl/fl*^) (Fig. 7e). Though the *Prx1-Cre;Penk*^*fl/fl*^ mice also showed no obvious differences in the adipocyte morphology under TN conditions (Supplementary Fig. S7b), decreased capacity in heat production under cold challenge was consistently observed (Fig. 7f).

Consistently, MetEnk was able to promote the expression of peroxisome proliferator-activated receptor-γ coactivator (*Pgc1α*) both in adipocyte precursor cells (Supplementary Fig. S7c) and mature adipocytes (Supplementary Fig. S7d), and the upregulation of *Pgc1α* by MetEnk was impaired in *Ogfr*-deficient adipocytes (Supplementary Fig. S7d).

Overall, those data support the important role of stromal MetEnk to adipocyte OGFr signal axis in regulating adipose heat production.

## Discussion

In this study, we identified and explored the important role of OGFr in regulating fatty acid metabolism and adipose tissue thermogenesis. As one of the most highly expressed neurotransmitter receptors in adipocytes, OGFr interacts MTPα, and promotes lipid dissipation and fatty acid oxidation. When *Ogfr* was ablated in adipocytes, the mice tended to accumulate lipids and display reduced thermogenic capacity, and developed more severe glucose intolerance and insulin insensitivity after chronic feeding with HFD. Meanwhile, the ligand for OGFr, MetEnk, can be derived from adipose stromal cells as its precursor encoding gene *Penk* was widely expressed in the stromal cell populations. Further, depletion of *Penk* in adipose stromal cells led to an impaired capacity of heat production. At the cellular level, the signal axis of MetEnk-OGFr enhanced cellular lipid utilization. The findings here have uncovered an uncharacterized signal pathway in mediating stromal-adipocyte intercellular interaction which promotes adipocyte energy expenditure and may be targeted to alter metabolic homeostasis.

Adipocytes have long been studied as the central player in the adipose tissues, whereas recently emerging evidence indicates that many of the tissue cell types may work coordinately to facilitate the processes of energy utilization or storage. The cell-to-cell communications between adipocytes, stromal cells, and other cells started to be recognized as crucial components in executing tissue function, however, our understanding remains far from complete. For instance, though the large collections of stromal cells compose the microenvironmental niche for the adipose depots, it is unknown how the stromal cells may be involved and regulated in the tissue activity. As one example following our study, it is unclear how the signal axis of MetEnk-OGFr is engaged between stromal cells and adipocytes, and further, how the production and release of the ligand are controlled. Previous studies on ILC2s show that the secretion of MetEnk is promoted by interleukin-33 (IL-33) [42] and could also be regulated by the sympathetic-dependent glial-derived neurotrophic factor from platelet derived growth factor receptor alpha positive mesenchymal cells [43]. It is thus intriguing to speculate that the comparable signaling events might also occur in stromal cells in stimulating the MetEnk-OGFr pathway. Nonetheless, future investigation in combination with experimental determination of cell–cell communications might offer further information on how the stromal cells and even additional cell types are differently and coherently engaged with adipocytes.

As a previously characterized neurotransmitter, the role of PENK has been shown in systemic inflammation, endocrine function, pain, memory, and reward [45, 65]. Given the increasingly recognized status of BAT as an endocrine organ [66, 67], the opioid-receptor axis may not be limited to the local effects. Instead, PENK-derived opioid ligands could likely be systemically involved in body functions, and potentially act on the opioid receptors expressed in other tissues like the brain [65]. And further, *Penk* is abundantly expressed in various fat pads which are distinctly activated by different metabolic stimuli, e.g., the ligands may be differentially available from either BAT, iWAT, or eWAT upon stimulations of cold, starvation, or energy surplus. Admittedly, the functions of PENK and OGFr could diverge based on their signaling targets, which was also indicated by our study here, as the stromal deletion of *Penk* seems not equal to adipocyte deletion of OGFr on the adipocyte sizes. In-depth studies are desirable to fully dissect their roles in various tissues or organs.

Our results suggest that OGFr alters cellular fuel utilization and the immediate question is how OGFr may promote lipid oxidation at the molecular level. Interestingly, OGFr has been detected both in the nucleus and the cytoplasm [34], and the translocation has been observed under the stimulation of MetEnk [68]. It would be, therefore, interesting to explore whether the locational change affects its function, such as direct involvement in transcription when present in the nucleus. Besides, NE and morphine have shown a synergistic effect in past studies [69–71], and a possible synergy might also exist between the sympathetic system and the opioid family, which awaits further exploration.

Previous studies have demonstrated the beneficial effects of brown/beige fat activation on metabolic health. The presence of BAT correlates with a lower likelihood of cardiometabolic disease [2]. Increased BAT activity induced by acute cold exposure or Adrb3 agonism in mice results in rapid uptake of fatty acids from triglyceride-rich lipoproteins and lowers the levels of circulating triglyceride and cholesterol [72, 73]. Since most adult humans have at least some BAT or beige fat [74, 75], a controlled increase in tissue activity seems a plausible approach. MetEnk has also been reported to effectively prevent body weight gain in mice treated with HFD through promoting browning of adipose tissue and can enhance glucose tolerance and insulin sensitivity, and was considered a potential therapy for metabolic disorders [76], but the underlying mechanisms are unclear. Given the inconvenience of cold challenge and side effects observed for Adrb3 agonists in humans [77–80], OGFr may serve as a potential therapeutic target for elevating the capacity of lipid consumption in adipose tissues. Nevertheless, *Ogfr* is widely expressed in different tissues and cell types, but is the most highly expressed opioid receptor in adipocytes. Based on the function of enkephalin in promoting beige cell formation, OGFr is probably the predominant receptor that respond to enkephalins in adipocytes. The advancement of local agent delivery technique may exploit the ligand-receptor interaction in fat and provides potential therapy for fat mobilization [81–83]. Identification of the regulatory mechanism for fat-burning activity remains viable for mitigating the deleterious effects of obesity such as hyperlipidemia associated with metabolic dysregulation.

## Materials and methods

### Antibodies and reagents

Antibodies used in this study were rabbit anti-GAPDH (Cell Signaling Technology Cat#5174, RRID:AB_10622025; rabbit anti-HSP90 (Cell Signaling Technology Cat# 4874, RRID:AB_2121214; rabbit anti-UCP1 (Abcam Cat# ab10983, RRID:AB_2241462; rabbit anti-Ace-Lys (Cell Signaling Technology Cat# 9814, RRID:AB_10544700; rabbit anti-HA (Sigma-Aldrich Cat# H6908, RRID:AB_260070; rabbit anti-MTP, (ABclonal Cat# A5346, RRID:AB_2863499; rabbit anti-MTP, (ABclonal Cat# A5716, RRID:AB_2766474; rabbit anti-HSL (Cell Signaling Technology Cat# 4107, RRID:AB_2296900); rabbit anti-pHSL (Ser563) (Cell Signaling Technology Cat# 4139, RRID:AB_2135495).

Reagents used in this study were CL-316,243 (Abcam, ab144605); MetEnk (Sigma, M6638); D(+)Glucose (Sigma, G7528); Dexamethasone (Sigma, D4902); IBMX (Sigma, I7018); Rosiglitazone (Sigma, R2408); Collagenase type I (Sigma, V900891); Oligomycin (Abcam, ab141829); FCCP (Sigma, C2920); 2-deoxy-d-glucose (2-DG) (Sigma, D8375); Rotenone (Sigma, R8875); Antimycin (Abcam, ab141904); Protease inhibitor (Bimake, B14001); Na_3_VO_4_ (Sigma, 450243); NaF (Sigma, 201154); β-Glycerophosphate (Sigma, G5422); PMSF (LABLEAD, 329-98-6); Glycine (Sigma, V900144); Tris Base (Sigma, V900483); SDS (Sigma, L5750); PA (Macklin, P815432); DHE (Beyotime, S0063); NE (ACMEC, CAS:108341-18-0); NEFA LabAssay (Wako, 294-63601); DMEM/F12 (Corning, 10-092); Seahorse XF Base Medium (Agilent, 102353-100); PrimeScript™ RT reagent kit with gDNA Eraser (Takara, RR047A); SYBR Green Master Mix (Vazyme, Q341-02); HiScribe™ T7 ARCA mRNA kit (NEB, E2060S); MEGAshortscript™ T7 High Yield Transcription Kit (Thermo Scientific, AM1354); Anti-HA magnetic beads (Thermo Scientific, 88836).

### Animal information

Mice were maintained on the 12 h:12 h light:dark cycle with the chow diet and water available *ad libitum* at 22°C. The control and mutant mice were in-house bred to produce the littermates for experiments. Both male and female mice maintained in specific pathogen-free conditions were utilized in the experiments. C57BL/6 mice were purchased from Tsinghua University. Gene expression and protein studies were performed on 6- to 12-week-old mice except for HFD-fed mice. HFD studies were conducted by feeding mice a purified ingredient standard diet (60% of calories from fat, 20% of calories from carbohydrate, and 20% of calories from protein; Research Diets) at 5 weeks of age. TN and cold exposure experiments were performed in climate-controlled rodent incubators maintained at 30°C or 4°C, respectively [84]. For TN experiments, mice were allowed to acclimate to 30°C for 10 days. For the acute cold challenge, mice were placed in prechilled cages at 4°C with padding, and free access to standard water, without food. No animals were excluded from studies and no randomization or blinding was performed.

*Penk*^*−/−*^, *Ogfr*^*fl/fl*^, and OGFr-HA knock-in (KI) mice were generated using Cas9-sgRNA system-mediated genomic deletion. sgRNAs (guide sequence, *Penk*^*−/−*^: 5ʹ-CCCTCTACAGACTATTCGGT-3ʹ, 5ʹ-CATCATTGGTGGAACCACGT-3ʹ; *Ogfr*^*fl/fl*^: 5ʹ-GGTAGTCAGGTTTGTAGTGC-3ʹ, 5ʹ-GCTCCATACTTAGTCTCTAT-3ʹ; OGFr-HA: 5ʹ-GAGGGGTACCTCTAAGGCTT-3ʹ) were delivered together with Cas9 mRNA [85] and DNA template (*Ogfr*^*fl/fl*^; OGFr-HA) into C57BL/6 mouse zygotes via microinjection in the in-house animal facility of Tsinghua University. The resulting littermates were screened by PCR genotyping and DNA sequencing. The *Penk*^*−/−*^ founder line with partial deletions on exon 3 (MGI:104629) was used for experiments. The *Ogfr*^*fl/fl*^ founder line with insertion of loxp sites before exon 2 and after exon 3 (MGI:1919325) was used for experiments. *Ogfr*^*fl/fl*^ was further crossed with *Adipoq-Cre* (RRID: IMSR_JAX:010803), *Ucp1-CreERT2* [48], or *CMV-Cre* mice (RRID: IMSR_JAX:006054) maintained on a C57BL/6J background. Age-matched littermates were subjected to experiments. Conditional *Penk* knockout mice were generated by mating *Prx1-Cre* (RRID: IMSR_JAX:005584) and *Penk*^*fl/fl*^ mice (Cyagen, S-CKO-04238). Age-matched littermates were subjected to experiments. The OGFr-HA founder line with insertion of HA tag into the C terminal of OGFr was used for experiments.

### HE staining and quantifications

For HE staining, the mice were euthanized and tissues were harvested and fixed in PBS/1% PFA at 4°C overnight, following by dehydration, clearing and infiltration, and microtomy. The slides were stained with standard HE staining protocol [84] and imaged by Nikon orthographic microscopic imaging system or Zeiss automatic digital slide scanning system. The size of adipocytes was counted and calculated by the softwares AdipoCount, Image pro plus, and Imaris.

### Culture of primary adipocytes and seahorse measurement

Primary white/beige adipocytes were prepared from the SVF isolated from iWAT. In brief, iWAT was isolated, minced finely, and digested in DMEM medium (Corning) containing 10% FBS, 10 mmol/L CaCl_2_, and Collagenase I (4 mg/mL) at 37°C for 30 min. After digestion, the SVF part cells were collected by centrifugation at 500 × *g* for 7 min and the red blood cells were removed with 1 × Ack lysis buffer for 1 min at RT. The cells were washed with DMEM medium containing 10% FBS and collected by centrifugation at 500 × *g* for 7 min, and then cultured in the maintaining DMEM/F12 medium (Corning) supplemented with 10% FBS and 1% penicillin-streptomycin. Adipocyte precursor cells were re-plated and differentiated with 850 nmol/L insulin, 0.5 μmol/L dexamethasone, 250 μmol/L IBMX, and 1 μmol/L rosiglitazone for 48 h when cells reached ~95% confluency. Cell culture was changed to DMEM/F12 medium plus 160 nmol/L insulin for another 48 h after induction and maintained in basic DMEM/F12 medium for 3 days. Mature adipocytes were examined for lipolysis assays or protein analysis.

For seahorse analysis, 10,000 SVF cells were plated onto each well of XF96 Cell Culture Microplates and the differentiation was inducted upon confluence. Fully differentiated adipocytes were used for seahorse analysis by XFe96 Extracellular Flux Analyzer. The OCR and ECAR were measured according to the manufacturer’s instructions. For OCR examination, the cells were preincubated in the absence of CO_2_ in Seahorse XF Base Medium (Agilent) containing 1 mmol/L l-Glutamine and 20 mmol/L glucose for at least 45 min at 37°C. The OCR was measured with the following reagents: 200 μmol/L PA, 1 μmol/L oligomycin, 1 μmol/L FCCP, 1 μmol/L rotenone, and 2 μmol/L antimycin A (Sigma). For ECAR examination, the cells were preincubated in the absence of CO_2_ in Seahorse XF Base Medium (Agilent) containing 1 mmol/L l-Glutamine for at least 45 min at 37°C. The ECAR was measured with the following reagents: 200 μmol/L PA, 20 mmol/L glucose, 1 μmol/L oligomycin, and 50 mmol/L 2-DG (Sigma).

### Lipolysis assay

For *in vivo* lipolysis assay, the mice were fasted overnight and injected intraperitoneally with isoproterenol (1 mg/kg body weight), and the plasma samples were collected before and after isoproterenol injection for 15 min. The serum total NEFA levels were determined by NEFA Lab Assay (Wako). The *in vitro* lipolysis assay was referred to the previous study [86]. In brief, fully differentiated adipocytes were preincubated with serum-free DMEM/F12 culture medium for 2 h and changed to KRBH buffer (30 mmol/L HEPES, 120 mmol/L NaCl, 4 mmol/L KH_2_PO_4_, 1 mmol/L MgSO_4_, 0.75 mmol/L CaCl_2_, and 10 mmol/L NaHCO_3_) with 2% fatty acid-free BSA and 5 mmol/L glucose. Then cells were fixed in PBS/1% PFA at RT for 10 min after stimulation with 1 μmol/L NE for 1–3 h. Cells were imaged with PerkinElmer Opera Phenix and the size of lipid droplets was quantified by PerkinElmer Opera Phenix related data analysis workstation.

### RNA isolation and gene expression analysis by qPCR

Mice were sacrificed by cervical dislocation and tissues were snap frozen in liquid nitrogen. Mouse tissues were homogenized in TRIzol and stored at −80°C. RNA was further extracted with chloroform ethyl alcohol and was reverse transcribed using PrimeScript™ RT reagent kit with gDNA Eraser (Takara). The cDNA was amplified by specific primers in a 20 μL reaction using SYBR Green (Vazyme) qPCR analysis. The following primer sequences were used for the mouse genes:

*Cyclophilin*-F, 5ʹ-TGGAGAGCACCAAGACAGACA-3ʹ; *Cyclophilin*-R, 5ʹ-TGCCGGAGTCGACAATGAT-3ʹ;

*Ucp1*-F, 5ʹ-GGAGAGAAACACCTGCCTCT-3ʹ; *Ucp1*-R, 5ʹ-ATTGTAGGTCCCCGTGTAGC-3ʹ;

*Pgc1α*-F, 5ʹ-CACCAAACCCACAGAAAACAG-3ʹ; *Pgc1α*-R, 5ʹ-GGGTCAGAGGAAGAGATAAAGTTG-3ʹ;

*Dio2*-F, 5ʹ-CGATTGATGTGGCTCCCTAAA-3ʹ; *Dio2*-R, 5ʹ-TCTGACTTTCTGCTTCGCTATC-3ʹ;

*Adipsin*-F, 5ʹ-CATGCTCGGCCCTACATGG-3ʹ; *Adipsin*-R, 5ʹ-CACAGAGTCGTCATCCGTCAC-3ʹ;

*Leptin*-F, 5ʹ-CAGGATCAATGACATTTCACACA-3ʹ; *Leptin*-R, 5ʹ-GCTGGTGAGGACCTGTTGAT-3ʹ;

*Adiponectin*-F, 5ʹ-GCACTGGCAAGTTCTACTGCAA-3ʹ; *Adiponectin*-R, 5ʹ-GTAGGTGAAGAGAACGGCCTTGT-3ʹ;

*Il1b*-F, 5ʹ-GCAACTGTTCCTGAACTCAACT-3ʹ; *Il1b*-R, 5ʹ-ATCTTTTGGGGTCCGTCAACT-3ʹ;

*Il10*-F, 5ʹ-CAGAGCCACATGCTCCTAGA-3ʹ; *Il10*-R, 5ʹ-TGTCCAGCTGGTCCTTTGTT-3ʹ;

*Cd11b*-F, 5ʹ-TCCGGTAGCATCAACAACAT-3ʹ; *Cd11b*-R, 5ʹ-GGTGAAGTGAATCCGGAACT-3ʹ;

*Penk*-F, 5ʹ-CTGAAAGAGCTACTGGGAACG-3ʹ; *Penk*-R, 5ʹ-ATACCTCTTGCTCATGTCTTCG-3ʹ;

*Ogfr*-F, 5ʹ-ATGACAAGGTACCGAAACTGG-3ʹ; *Ogfr*-R, 5ʹ-TCCGTTGCAGTCTTGATCTG-3ʹ;

*Ccl2*-F, 5ʹ-CTCGGACTGTGATGCCTTAAT-3ʹ; *Ccl2*-R, 5ʹ-TGGATCCACACCTTGCATTTA-3ʹ;

*iNos*-F, 5ʹ-CAGCTGGGCTGTACAAACCTT-3ʹ; *iNos*-R, 5ʹ-CATTGGAAGTGAAGCGTTTCG-3ʹ;

*Tnfα*-F, 5ʹ-CCAAGGCGCCACATC TCCCT-3ʹ; *Tnfα*-R, 5ʹ-GCTTTCTGTGCTCATGGTGT-3ʹ;

*Fgf21*-F, 5ʹ-CTACACAGATGACGACCAAGAC-3ʹ; *Fgf21*-R, 5ʹ-CTTTGAGCTCCAGGAGACTTTC-3ʹ;

*Sod1*-F, 5ʹ-CTCAGGAGAGCATTCCATCATT-3ʹ; *Sod1*-R, 5ʹ-CTCCCAGCATTTCCAGTCTT-3ʹ;

*Sod2*-F, 5ʹ-CAGACCTGCCTTACGACTATG-3ʹ; *Sod2*-R, 5ʹ-GTGGCGTTGAGATTGTTCAC-3ʹ;

*Cox2*-F, 5ʹ-CCTCGTCCAGATGCTATCTTTG-3ʹ; *Cox2*-R, 5ʹ-GGCTTCCAGTATTGAGGAGAAC-3ʹ;

*Nqo1*-F, 5ʹ-GAGAAGAGCCCTGATTGTACTG-3ʹ; *Nqo1*-R, 5ʹ-ACCTCCCATCCTCTCTTCTT-3ʹ;

*Nox4*-F, 5ʹ-CCAGAATGAGGATCCCAGAAAG-3ʹ; *Nox4*-R, 5ʹ-GGTAGAAGCTGTAACCATGAGG-3ʹ;

*Duox1*-F, 5ʹ-GGCCAGCATCTCCTTTATGT-3ʹ; *Duox1*-R, 5ʹ-AAGGGAATGTAGCGGTTGAG-3ʹ;

*Cd206*-F, 5ʹ-CCACAGCATTGAGGAGTTTG-3ʹ; *Cd206*-R, 5ʹ-ACAGCTCATCATTTGGCTCA-3ʹ;

*Oprd1*-F, 5ʹ-CATCGTCCGGTACACCAAAT-3ʹ; *Oprd1*-R, 5ʹGGCCACGTTTCCATCAAGTA-3ʹ;

*Oprk1*-F, 5ʹ-GGGACTTCTGCTTCCCTATTAAG-3ʹ; *Oprk1*-R, 5ʹ-CTTATTCATCCCTCCCACATCTC-3ʹ;

*Oprl1*-F, 5ʹ-CGGTCATTGCTATCGACTACTAC-3ʹ; *Oprl1*-R, 5ʹ-AACATCAAGGGCACGGATAG-3ʹ;

*Oprm1*-F, 5ʹ-GACTGTTTCCTGGCACTTCT-3ʹ; *Oprm1*-R, 5ʹ-GTTGGGATGCAGAACTCTCTAA-3ʹ.

### Protein extraction and immunoblot analysis

Mice were euthanized and tissues were snap frozen in liquid nitrogen. Mouse tissues were homogenized in RIPA buffer (50 mmol/L Tris-Cl, 150 mmol/L NaCl, 0.5% sodium deoxycholate, 0.1% SDS, 1% Triton X-100, 1 mmol/L Na_3_VO_4_, 0.1 mmol/L NaF, 20 mmol/L β-glycerophosphate, and 1 mmol/L PMSF) and the protein concentration was measured with BCA kit. Cultured cells were washed by PBS buffer and lysed in RIPA buffer. The samples were then boiled at 95°C in 2 × SDS loading buffer and western blotting was carried out using standard protocols. Blots were blocked for 1 h with 5% skim milk in TBST (1 × TBS with 0.1% Tween-20) and were incubated overnight at 4°C with primary antibodies. Blots were washed three times in TBST for 10 min, then incubated with HRP-conjugated secondary antibodies for 1 h at RT, washed and visualized using chemiluminescence (Thermo Scientific), and quantified by Image J.

### Immunoprecipitation assays

Mice were sacrificed by cervical dislocation, and tissues were collected in IP lysis buffer (50 mmol/L Tris-Cl, pH7.4, 150 mmol/L NaCl, 10% glycerol, 1% Triton X-100, and protease inhibitor cocktail) and transferred into homogenizer and homogenized for 20 times. The supernatant was kept after centrifugation for 20 min at 15,000 × rpm and incubated with anti-HA beads at 4°C for 2 h. The magnetic beads were washed with washing buffer (50 mmol/L Tris-Cl, pH7.4, 150 mmol/L NaCl, 10% glycerol, 0.1% Triton X-100, and protease inhibitor cocktail) for 3 times and proteins were eluted in 2 × SDS loading buffer at 95°C for 10 min. Western blotting and mass spectrum were carried out using standard protocols. For HeLa cell immunoprecipitation, cells were washed with cold PBS for 3 times and collected in IP lysis buffer (50 mmol/L Tris-Cl, pH 7.4, 150 mmol/L NaCl, 10% glycerol, 1% Triton X-100, and protease inhibitor cocktail) and the supernatant was kept after centrifugation for 20 min at 15,000 × rpm and incubated with anti-HA beads at 4°C for 2 h. The magnetic beads were washed with washing buffer (50 mmol/L Tris-Cl, pH 7.4, 150 mmol/L NaCl, 10% glycerol, 0.5% Triton X-100, and protease inhibitor cocktail) for 3 times and proteins were eluted in 2 × SDS loading buffer at 95°C for 10 min. Western blotting was carried out using standard protocols.

### Fluorescent immunohistochemistry

For the fluorescent immunohistochemistry, cells were fixed in PBS/1% PFA at RT for 10 min, then washed with PBS, immunolabeled with indicated primary antibodies and corresponding Alexa dye-conjugated secondary antibodies, and imaged by fluorescence microscopy Zeiss LSM 980, analyzed by related software.

### Cell fractionation

HeLa cells were washed by PBS buffer and homogenized in hypotonic buffer (10 mmol/L Tris-Cl, pH7.4, 10 mmol/L KCl, 0.5 mmol/L EGTA, 1.5 mmol/L MgCl_2_, and EDTA-free protease inhibitor cocktail). The homogenates were then centrifuged at 1000 × *g* for 5 min to pellet nuclei and unbroken cells (P1) and the supernatant was collected as S1.

### *In vivo* metabolic phenotyping and whole-animal OCR

The whole-body metabolism activities were evaluated by the CLAMS system at RT. Mice were allowed to be acclimated in metabolic chambers for 1 day before data collection. For the oxygen consumption test, mice were housed at 22°C and anaesthetized with 375 mg/kg tribromoethanol, and baseline oxygen consumption was recorded for several cycles. Then 1 mg/kg NE dissolved in saline was administered intraperitoneally and NE-induced oxygen consumption was measured until the rates began to decline [87].

### Core body temperature measurement and cold-tolerance test

The core body temperatures were measured by IPTT-300 Programmable Temperature Transponder (BMDS). The transponder was injected beneath the dorsal nuchal region 2 days before the cold challenge. For the acute cold challenge, mice were allowed to acclimate to 30°C for 10 days, and were placed in prechilled cages at 4°C with padding, free access to standard water, without food. Temperatures were recorded every 30 min and the mice were sacrificed when their body temperatures were below 28°C.

### Glucose tolerance and insulin sensitivity tests

For OGTT, mice were fasted for 16 h overnight and gavaged with glucose (2 g/kg body weight for HFD-fed mice). Blood glucose was determined by GA-3 glucometer (Sinocare) at different time points. For IPGTT, mice were fasted for 16 h overnight and injected intraperitoneally with glucose (1 g/kg body weight for chow-diet fed mice). Blood glucose was determined by GA-3 glucometer at different time points. For ITT, mice were fasted for 2–5 h following injection with 0.75 U/kg body weight of recombinant human insulin, and blood glucose was determined by GA-3 glucometer at different time points.

### Sample preparation for serum metabolite examination

For serum collection, mice were anesthetized, and blood was taken from the orbit, placed at RT for more than 1 h or 4°C for 2 h, and then centrifuged at 3000 × rpm for 10 min. The supernatant serum was collected and stored at −80°C for further examination. The serum total NEFA levels were determined by NEFA Lab Assay (Wako). For fatty acids examination preparation, 50 μL serum was transferred to a new tube. Methanol (MeOH), methyl tert-butyl ether (MTBE), and water were added according to the volume ratio MeOH/MTBE/H_2_O (1:5:1.5 v:v:v), vortexed for 1 min, and centrifuged at 14,000 × *g* for 10 min at 4°C. The supernatant was further collected and N_2_ was used to dry the pellet (using no heat). The samples were examined by Q Exactive (Thermo Scientific) at Metabolomics and Lipidomics Center, Tsinghua University. For acyl-carnitines examination preparation, 50 μL serum was transferred to a new tube and 200 μL prechilled MeOH was added to the serum to make a final 80% (v/v) MeOH solution. The sample was gently mixed and incubated at −80°C overnight, and then centrifuged at 14,000 × *g* for 10 min at 4°C. The 200 μL supernatant was collected in a new tube and dried under vacuum, and the dried samples were examined by Q Exactive HF-X (Thermo Scientific) at Metabolomics and Lipidomics Center, Tsinghua University.

### scRNA-Seq and snRNA-Seq analysis

We used Seurat v4.0.1 [88], and analyzed with R version 4.0.4 for single-cell transcriptomic data. The scRNA-Seq results of mouse adipose tissue were collected from GSE128889 (GSM3717977) [47]. We reanalyzed the gene expression levels using data filtered by nFeature between 350 and 7000, and the dim character was 1:18 during the clustering algorithm. The cell classification was consistent with the literature. The t-distributed stochastic neighbor embedding (t-SNE) and GraphPad were used to visualize the datasets. The snRNA-Seq results of human adipose tissue were collected from GSE176171 (GSM5359331, GSM5359332, GSM5359333, GSM5359334, GSM5359335, and GSM5820689) [31]. We reanalyzed the gene expression levels using data filtered by nFeature between 350 and 7000, and the dim character was 1:17 during the clustering algorithm. The cell classification was consistent with the literature. The t-SNE and GraphPad were used to visualize the datasets.

### Quantification and statistical analysis

The data were analyzed with Graphpad Prism 8 in the website of Graphpad. For the comparisons of the two groups, statistical analyses were performed using unpaired two-tailed Student’s *t* test. Welch’s correction was used when the variances of the samples were unequal. For the comparisons of time course data among two or more groups, two-way ANOVA was applied. For the survival rate, Mantel-Cox test was applied. The sample capacity can be found in the figure legends. Each *n* represents the number of mice and is indicated in the figure legends. *P <* 0.05 was considered significant. No statistical methods were used to predetermine sample size. ^*^P < 0.05, ^**^P < 0.01, ^***^P < 0.001, ^****^P < 0.0001, n.s., not significant. Error bars represent SEM.

## Supplementary Material

load018_suppl_Supplementary_Figures

## Data Availability

Further information and requests for resources and reagents should be directed to and will be fulfilled by the Lead Contact, Wenwen Zeng (wenwenzeng@tsinghua.edu.cn).
