# Supplementary material for: Opioid growth factor receptor promotes adipose tissue thermogenesis via enhancing lipid oxidation
Source: Life Metab. 2023 May 4;2(3):load018. doi: 10.1093/lifemeta/load018 (PMC11749475; doi:10.1093/lifemeta/load018)
Supplement: load018_suppl_Supplementary_Figures [file load018_suppl_Supplementary_Figures.docx]

Supplementary Materials for

**Opioid growth factor receptor promotes adipose tissue thermogenesis via enhancing lipid oxidation**

Shan Zhang, Jianhui Chen, Qingqing Li, Wenwen Zeng

*Corresponding author. Email: [wenwenzeng@tsinghua.edu.cn](mailto:xxxxx@xxxx.xxx)

**This PDF file includes:**

Figs. S1 to S7


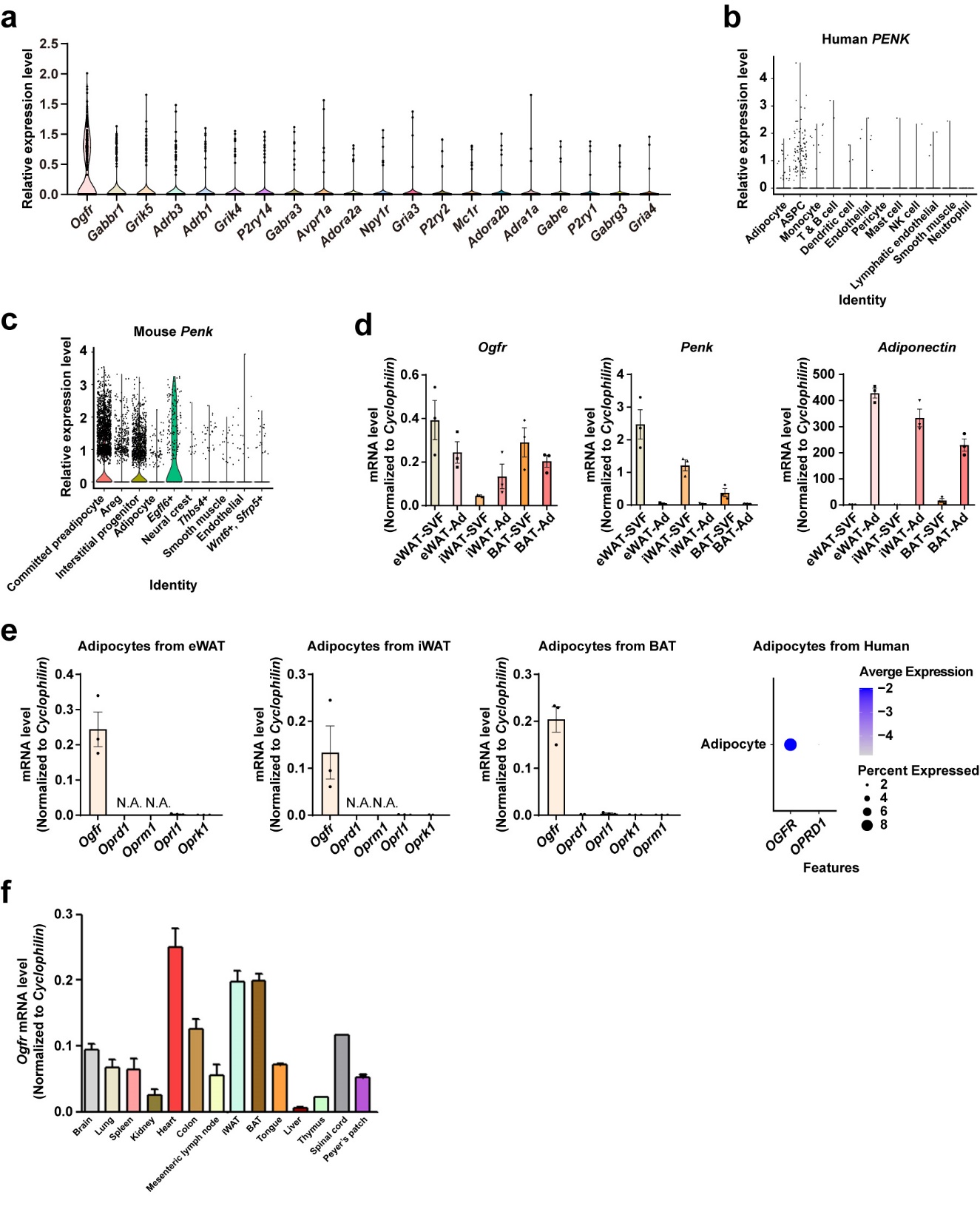


# Fig. S1 (related to Fig. 1) *Ogfr* is highly expressed in adipocytes and positively regulates energy consumption. (a) Individual gene tSNE and violin plots showing the expression levels of neurotransmitter receptors from mouse scRNA-Seq results in adipocytes. (b) Individual gene tSNE and violin plots showing the expression levels of *PENK* from human sn-RNA-Seq results in different cell types in WAT. ASPC, adipose stem and progenitor cell. (c) Individual gene tSNE and violin plots showing the expression levels of *Penk* from mouse scRNA-Seq results in different cell types in WAT. Areg, adipogenesis-regulatory cell; *Egfl6*, epidermal growth factor-like domain 6; *Thbs4*, thrombospondin 4; *Sfrp5*, secreted frizzled-related protein 5. (d) qPCR determination of *Ogfr*, *Penk*, and *Adiponectin* expression in isolated mature adipocytes and SVF of interscapular BAT, iWAT and eWAT from wild-type mice. n=3. Female. Mean ± SEM. (e) qPCR determination of opioid receptors expression in adipocytes isolated from BAT and WAT in wild-type mice. n=3. Female. Mean ± SEM. N.A., not available. And expression levels of opioid receptors from human snRNA-Seq results in adipocytes, *OPRK1*, *OPRL1* and *OPRK1* not available. (f) qPCR determination of *Ogfr* expression in different tissues from wild-type mice. n=2. Female. Mean ± SEM.


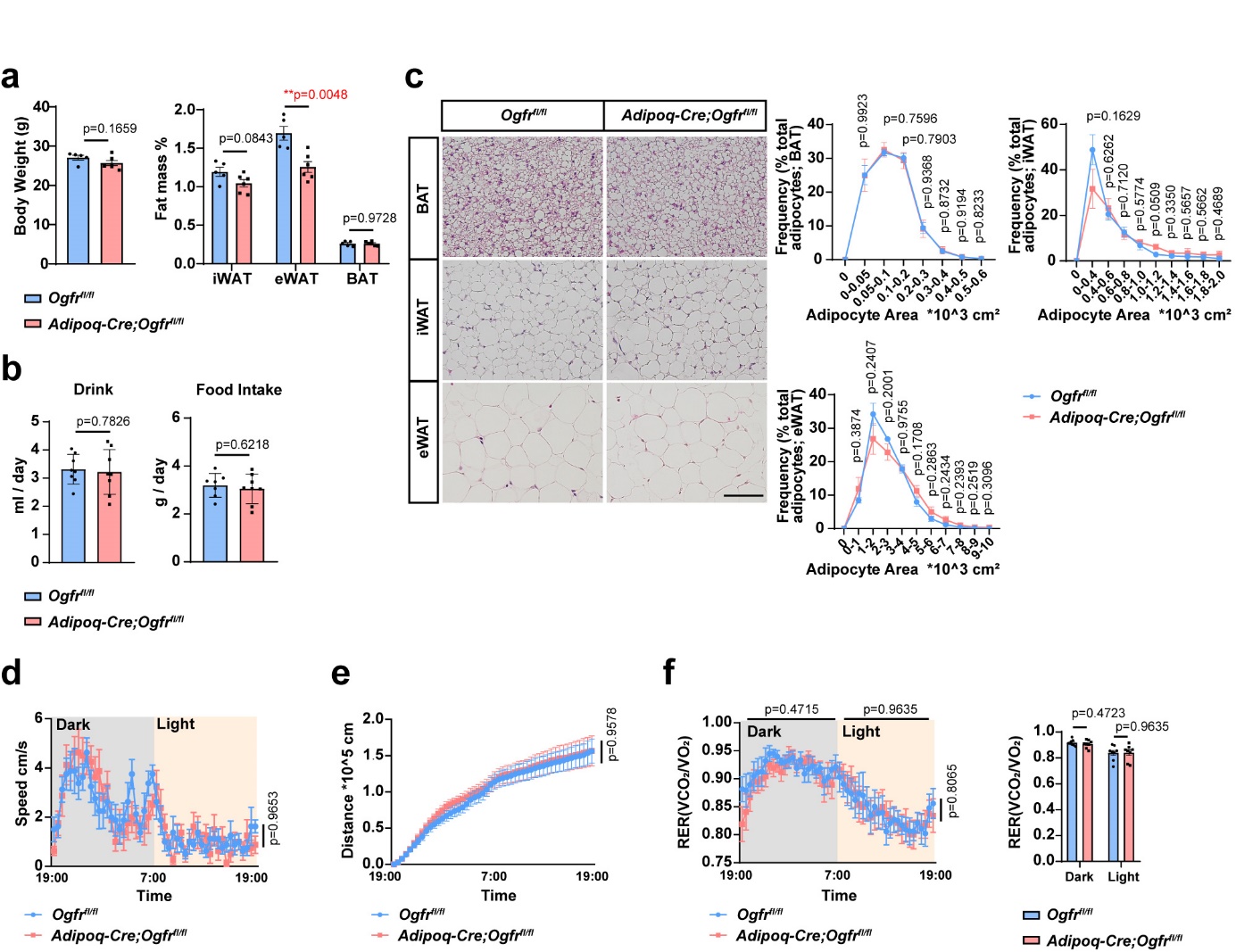


Fig. S2 (related to Fig. 1) *Ogfr* is highly expressed in adipocytes and positively regulates energy consumption. (a) Body weight and fat mass (normalized to body weight) of *Adipoq-Cre;Ogfr^fl/fl^* mice and control littermates housed at 22℃. For *Adipoq-Cre;Ogfr^fl/fl^*, n=6. For *Ogfr^fl/fl^*, n=5. Male. Mean ± SEM; **p＜0.01 by Student’s t test. (b) The CLAMS metabolic cage analysis of drink and food intake of *Adipoq-Cre;Ogfr^fl/fl^* mice and control littermates housed at 22℃. n=8. Female. Mean ± SEM; Student’s t test. (c) HE staining and quantifications of interscapular BAT, iWAT, and eWAT from *Adipoq-Cre;Ogfr^fl/fl^* mice and control littermates housed at 22℃. Scale bar, 100 μm. For BAT, n=3, male. For iWAT, n=4 for *Adipoq-Cre;Ogfr^fl/fl^* mice, n=5 for *Ogfr^fl/fl^* mice, male. For eWAT, n=4, female. Mean ± SEM; Student’s t test. (d) The CLAMS metabolic cage analysis of speed of *Adipoq-Cre;Ogfr^fl/fl^* mice and control littermates housed at 22℃. n=8. Female. Mean ± SEM; unmatched two-way ANOVA test. (e) The CLAMS metabolic cage analysis of move distance of *Adipoq-Cre;Ogfr^fl/fl^* mice and control littermates housed at 22℃. n=8. Female. Mean ± SEM; unmatched two-way ANOVA test. (f) The CLAMS metabolic cage analysis of RER of *Adipoq-Cre;Ogfr^fl/fl^* mice and control littermates housed at 22℃. n=8. Female. Mean ± SEM; unmatched two-way ANOVA test for curve and Student’s t test for histogram.


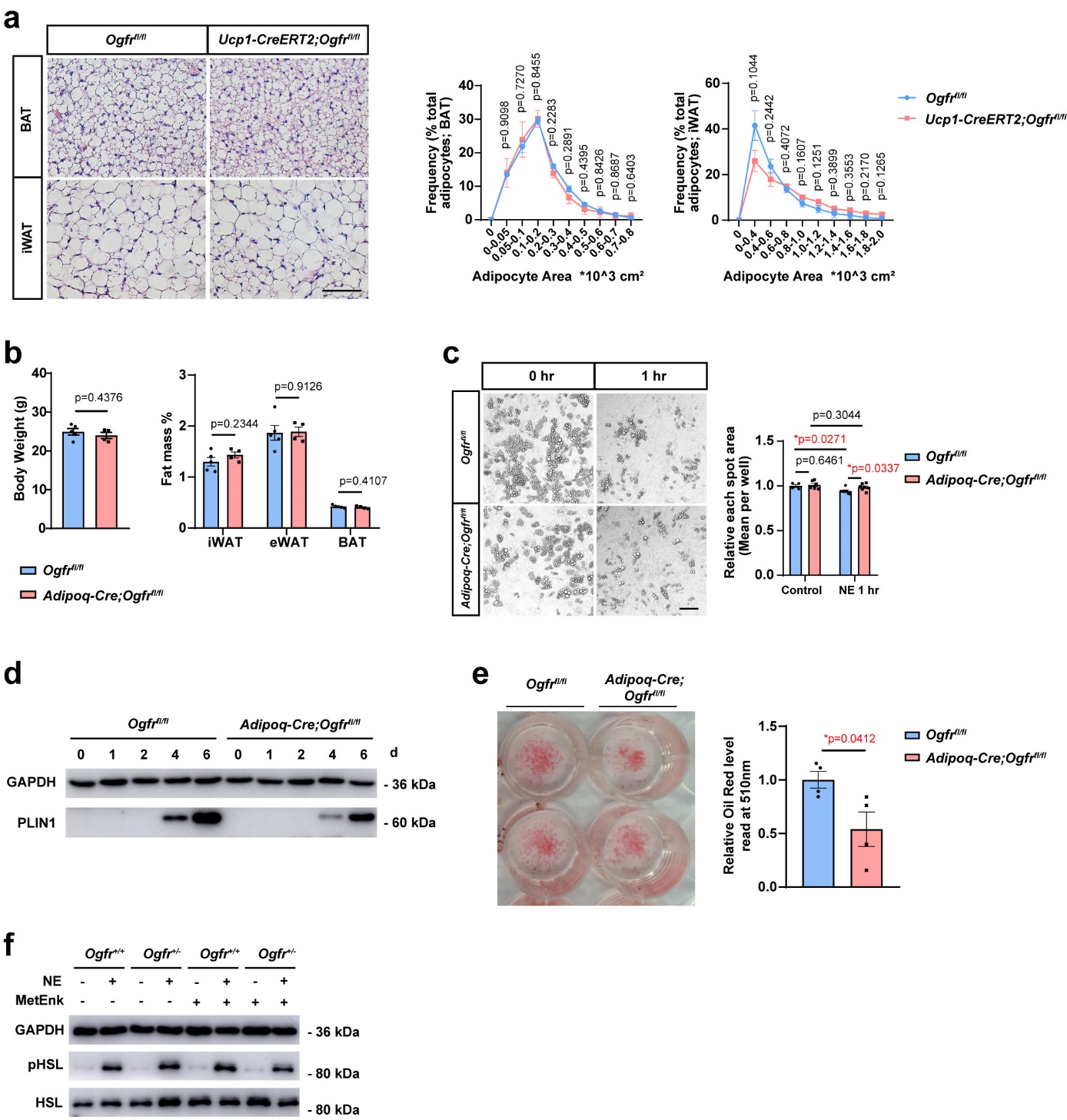


Fig. S3 (related to Fig. 3) OGFr enhances adipocyte lipid utilization for heat generation in response to cold exposure. (a) HE staining and quantifications of interscapular BAT and iWAT from *Ucp1-CreERT2;Ogfr^fl/fl^* mice and control littermates housed at 30℃. Scale bar, 100 μm. n=4. Female. Mean ± SEM; Student’s t test. (b) Body weight and fat mass (normalized to body weight) of *Adipoq-Cre;Ogfr^fl/fl^* mice and control littermates housed at 30℃. For *Adipoq-Cre;Ogfr^fl/fl^*, n=4. For *Ogfr^fl/fl^*, n=5. Male. Mean ± SEM; Student’s t test. (c) *In vitro* lipolysis analysis and quantified spot size of cultured primary adipocytes from *Adipoq-Cre;Ogfr^fl/fl^* mice and control littermates, treated with 1 μM NE for 0-1 hr. Scale bar, 100 μm. Mean ± SEM; *p＜0.05 by Student’s t test. (d) Immunoblot analysis of GAPDH and perilipin 1 (PLIN1) in primary adipocytes cultured from *Adipoq-Cre;Ogfr^fl/fl^* mice and control littermates during differentiation at 0,1,2,4,6 days. (e) The image and oil red levels in primary mature adipocytes cultured from *Adipoq-Cre;Ogfr^fl/fl^* mice and control littermates. n=4. *p＜0.05 by Student’s t test. (f) Immunoblot analysis of GAPDH, pHSL and HSL of cultured primary adipocytes from *Ogfr^+/-^* mice and control littermates, treated with 1 μg/ml MetEnk or 0.1 μM NE stimulation for 30 min.


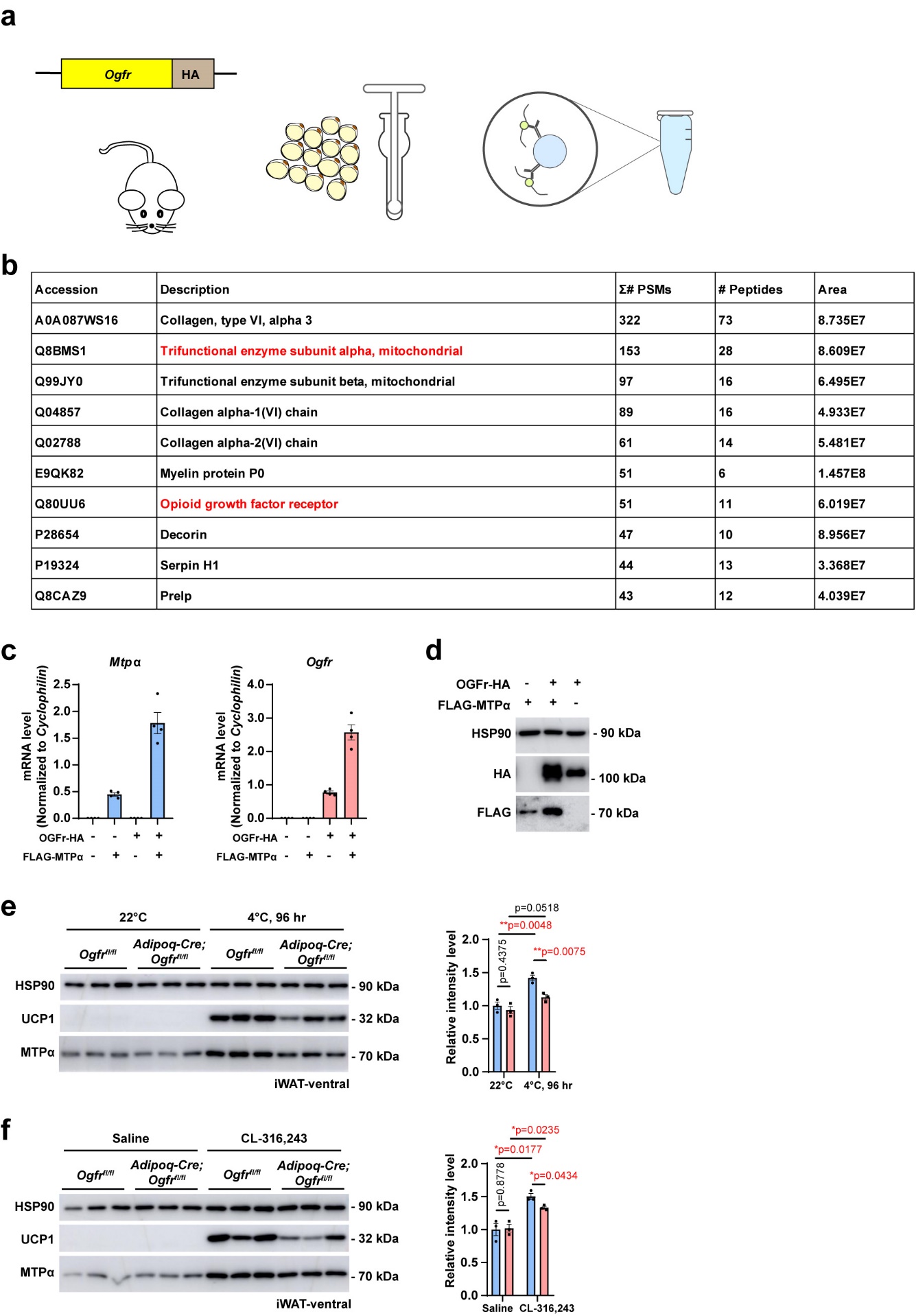


Fig. S4 (related to Fig. 4) OGFr interacts with MTPα. (a) Strategy of OGFr-HA KI mice and immunoprecipitation with anti-HA of iWAT. (b) The list of top 10 hits identified in HA group from mass spectrometric analysis. (c) qPCR analysis of *Mtpα* and *Ogfr* gene expression changes in overexpressed 293T cells. n=4. (d) Immunoblot analysis of OGFr-HA and FLAG-MTPα in overexpressed 293T cells. (e) UCP1 and MTPα immunoblot analysis of iWAT from *Adipoq-Cre;Ogfr^fl/fl^* mice and control littermates housed at 22℃ and cold challenged for 96 hr at 4℃. n=3. Male. Mean ± SEM; *p＜0.05, **p＜0.01 by Student’s t test. (f) UCP1 and MTPα immunoblot analysis of iWAT from *Adipoq-Cre;Ogfr^fl/fl^* mice and control littermates with intraperitoneal saline or CL-316,243 treatment for 4 days at 22℃. n=3. Female. Mean ± SEM; *p＜0.05 by Student’s t test.


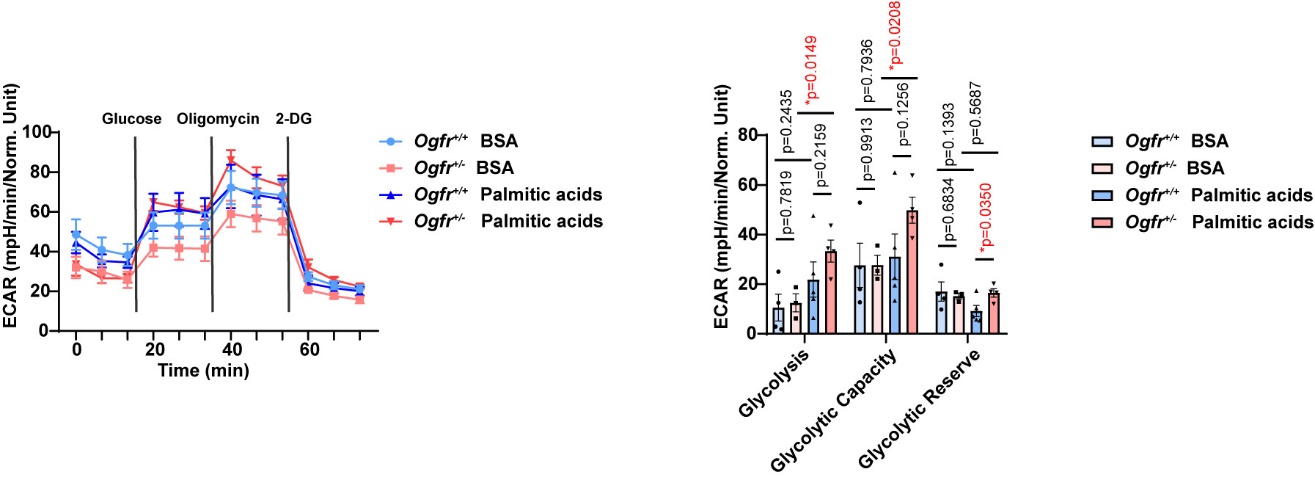


Fig. S5 (related to Fig. 5) OGFr enhances adipocyte fatty acid oxidation. ECAR analysis of cultured primary adipocytes from *Ogfr^+/-^* mice and control littermates. Treated with BSA or 200 μM palmitic acids, followed with glucose (20 mM), oligomycin (1 μM), 2-Deoxy-D-glucose (2-DG) (50 mM) as indicated. For *Ogfr^+/-^* cells*,* n=3 for BSA group and n=4 for palmitic acids group. For control cells, n=4 for BSA group and n=5 for palmitic acids group. Female. Mean ± SEM; *p＜0.05 by Student’s t test.

**
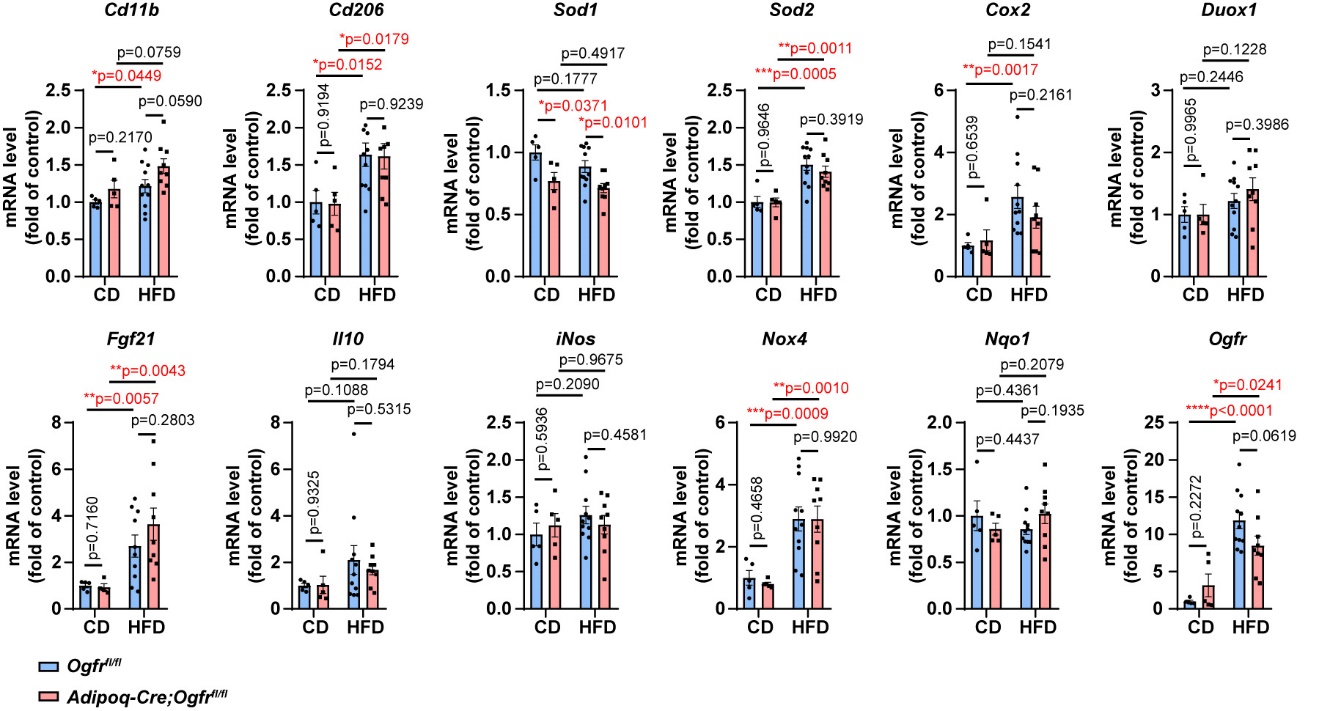
**

**Fig. S6 (related to Fig. 6) OGFr protects from high-fat diet induced glucose intolerance.** qPCR analysis of oxidative and inflammatory genes expression in eWAT from *Adipoq-Cre;Ogfr^fl/fl^* mice versus control littermates fed chow- or HFD for 18 weeks at 22℃. For *Adipoq-Cre;Ogfr^fl/fl^*, n=5 for chow diet and n=9 for HFD. For *Ogfr^fl/fl^*, n=5 for chow diet and n=11 for HFD. Mean ± SEM; *p＜0.05, **p＜0.01, ***p＜0.001, ****p＜0.0001 by Student’s t test.


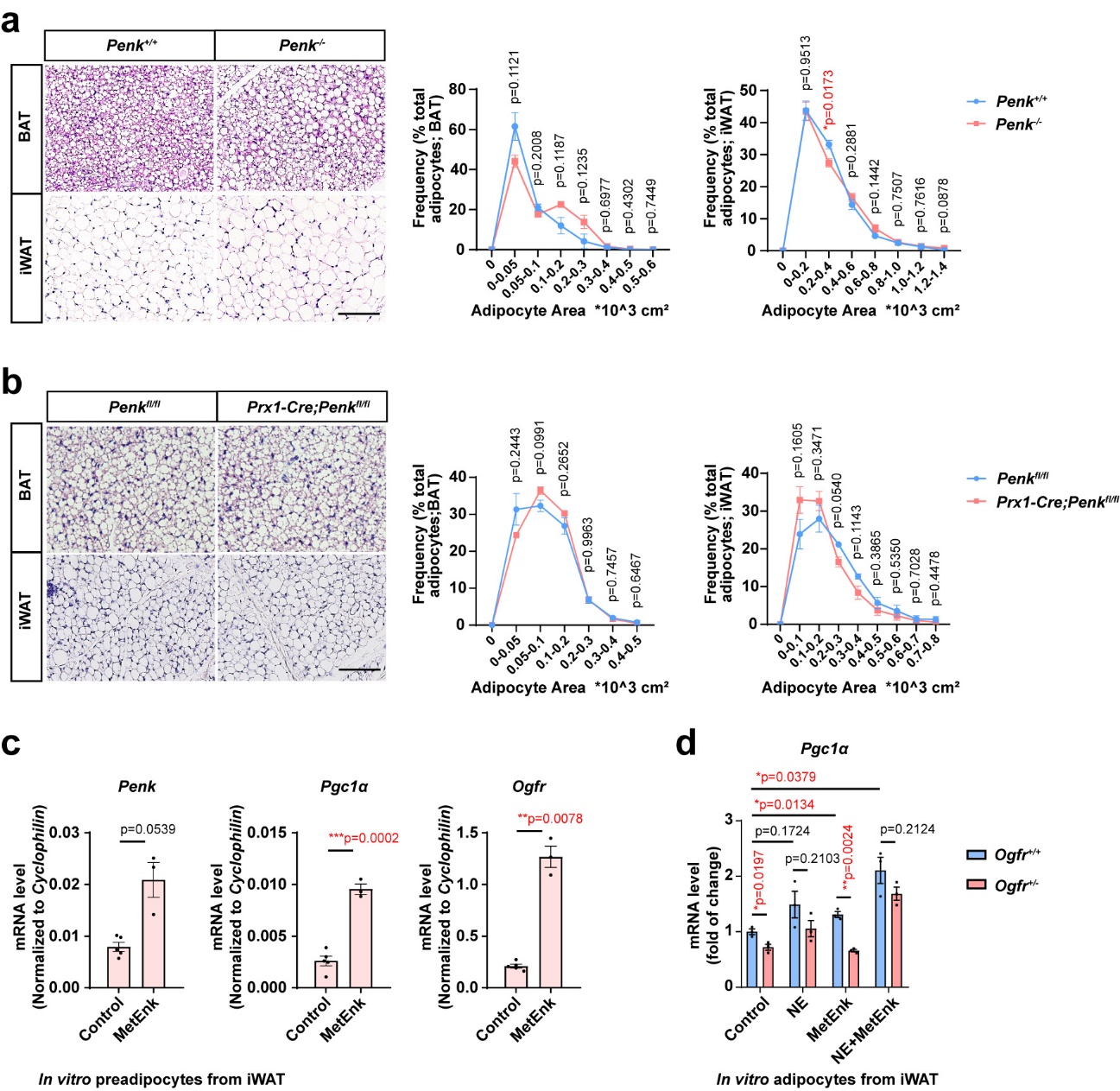


Fig. S7 (related to Fig. 7) Stromal MetEnk to adipocyte OGFr axis enhances thermogenesis in response to cold exposure. (a) HE staining and quantifications of interscapular BAT and iWAT from *Penk^-/-^* mice and control littermates housed at 30℃. Scale bar, 100 μm. For BAT, n=3, female. For iWAT, n=5 for *Penk^-/-^* mice and n=4 for control mice, male. Mean ± SEM; *p＜0.05 by Student’s t test. (b) HE staining and quantifications of interscapular BAT and iWAT from *Prx1-Cre;Penk^fl/fl^* mice and control littermates housed at 30℃. Scale bar, 100 μm. n=3. Female. Mean ± SEM; Student’s t test. (c) qPCR verification of *Penk*, *Pgc1α,* and *Ogfr* gene expression in adipocyte precursor cells from wild-type mice treated with 1 μg/mL MetEnk for 1 hr. For MetEnk group, n=3. For control group, n=5. Female. Mean ± SEM; **p＜0.01, ***p＜0.001 by Student’s t test. (d) qPCR verification of *Pgc1α* gene expression in cultured primary mature adipocytes from *Ogfr^+/-^* and control mice, treated with 0.1 μM NE or 1 μg/mL MetEnk for 1 hr. n=3. Mean ± SEM; *p＜0.05, **p＜0.01 by Student’s t test.
